# Eye-Tracking in Bipolar Disorder: A Methodology-Focused Narrative Review

**DOI:** 10.3390/medicina62091626

**Published:** 2026-08-24

**Authors:** Evrim Bayrak Oruc, Buket Koparal, Jonathan Jacobs, Aasef G. Shaikh, Keming Gao

**Affiliations:** 1 Department of Psychiatry, University Hospitals Cleveland Medical Center, Cleveland, OH 44106, USA; evrim.bayrakoruc@uhhospitals.org; 2 Department of Psychiatry, Gazi University School of Medicine, Ankara 06500, Turkey; 3 Department of Psychiatry, Case Western Reserve University School of Medicine, Cleveland, OH 44106, USA; 4 Department of Neurology, University Hospitals, Cleveland, OH 44106, USA; 5 Department of Neurology, Case Western Reserve University School of Medicine, Cleveland, OH 44106, USA; 6 Daroff-Dell’Osso Ocular Motility Lab, Cleveland VA Medical Center, Cleveland, OH 44106, USA; 7 Department of Biomedical Engineering, Case Western Reserve University, Cleveland, OH 44106, USA; 8 Functional Electrical Stimulation Center, Cleveland VA Medical Center, Cleveland, OH 44106, USA; 9 Department of Psychosomatic Medicine, Sichuan Lansheng Brain Hospital, Chengdu 610036, China

**Keywords:** bipolar disorder, eye-tracking, free viewing task, antisaccade, prosaccade, memory guided saccade, binocular rivalry, smooth pursuit eye movements, vergence, eye blink rate

## Abstract

*Background and Objectives:* This methodology-focused narrative review synthesizes the eye-tracking literature in bipolar disorder (BD) to evaluate the utility of such technology in identifying potential state or trait biomarkers for BD. *Methods:* A PubMed and Scopus search was performed from database inception to August 2026. Eligible studies included adults with BD or individuals at high-risk for BD compared with healthy controls (HCs) that used at least one quantitative eye-tracking paradigm and reported extractable quantitative outcomes. Studies were grouped into eight paradigms: free-viewing task (FVT), antisaccade (AS), prosaccade (PS), memory-guided saccade (MGS), smooth pursuit eye movements (SPEM), vergence, binocular rivalry (BR), and eye-blink rate (EBR). Findings were synthesized narratively based on study designs and outcome measures. *Results:* 24 studies met inclusion criteria. FVT and PS were the most frequently represented paradigms (*n* = 8 each), followed by AS (*n* = 7), BR (*n* = 4), and SPEM (*n* = 3); MGS, vergence, and EBR were each represented by one report. Findings from FVT, AS, and PS were inconsistent; the study designs, stimulus types, mood states during the study, and outcome measures of studies with these paradigms were diverse. BR alternation rate was consistently slower in BD across three studies. SPEM deficits were more closely associated with psychotic features. Evidence from single studies of MGS, vergence, and EBR limits their interpretations. *Conclusions:* Eye-tracking paradigms are promising tools for studying cognitive, affective, perceptual, sensorimotor, and reward-related presentations of BD. However, the field needs standardized protocols that include eye-tracking paradigms, mood states, symptom severity, outcome measures, study samples, and comparison groups to examine the utility of eye-tracking technology in BD.

## 1. Introduction

Bipolar disorder (BD) affects approximately 1–2% of people over their lifetime, and it is among the leading causes of disability worldwide [1]. Despite its prevalence and clinical impact, BD is associated with an average diagnostic delay of 6–10 years, frequent misdiagnosis as unipolar depression, and a heterogeneous treatment response [2,3]. A major challenge is the absence of objective, measurable markers that can support diagnosis, stage illness, and monitor treatment responses [4]. Biomarkers that can distinguish state-dependent fluctuations, which reflect the current mood episode, from trait-like vulnerabilities that persist across mood states would substantially improve the clinical management of BD [5,6]. Ocular-motor measurement has emerged as one candidate approach for this purpose [7,8].

Eye tracking offers objective, millisecond-level measurement of eye movements, which may reflect underlying cognitive and affective processes without depending on self-reports [9,10]. In BD, self-reports may be unreliable due to impaired insight during a manic episode or stigma associated with a diagnosis of BD in a euthymic period. Meanwhile, recall bias during mood episodes, especially during depressive episodes, may occur and possibly cause underreporting of manic/hypomanic symptoms [11]. These intentional and unintentional underreportings likely cause misdiagnoses of BD as major depressive disorders or psychotic disorders.

Eye-tracking paradigms can measure distinct elements of information processing, such as inhibitory control, attention distribution, reward-based orienting, and predictive motor tracking. Each element corresponds to distinct neural circuits that are likely involved in the pathophysiology of BD [10,12]. This dimensional approach aligns with the Research Domain Criteria (RDoC) framework, which conceptualizes that psychiatric symptoms are correlated to different underlying neurobiological systems rather than categorical diagnoses [13]. Because BD is characterized by clinically different mood states, different neuronal networks may be involved across manic, depressive and euthymic episodes. Since eye-tracking paradigms can probe the functions of different networks, eye-tracking technology may help us understand the changes in functions and symptom presentations during the different mood states of BD.

Previous reviews have focused on circumscribed aspects of ocular-motor function in BD. Carvalho et al. (2015) reviewed eye-movement findings across unipolar and bipolar depression, focusing on prosaccade and antisaccade reaction time and attentional bias to emotional stimuli [7]. Miskowiak et al. (2019) examined affective cognition in BD across emotional face processing, emotion regulation, and reward processing, with eye-tracking discussed only within these affective domains [14]. De Prisco et al. (2025) conducted a systematic review and meta-analysis of image-viewing eye-tracking metrics in BD, restricted to fixation latency, fixation count, gaze duration, and saccadic peak velocity and amplitude [15]. However, BD involves disturbances across several cognitive and affective domains, including emotion regulation, attentional control, executive functioning, and reward sensitivity [16]. Because each eye-movement paradigm captures only a restricted component of these processes, no single paradigm can adequately represent the full range of dysfunction associated with BD. In addition, several paradigms, including binocular rivalry, vergence, and eye-blink rate, have received relatively little attention in the BD review literature despite their potential relevance to perceptual, sensorimotor, and dopaminergic dysfunction.

No prior review has synthesized findings across the full set of eye-movement paradigms applied in BD. This review provides a broad, methodology-focused synthesis of eight eye-movement paradigms in BD that includes free-viewing task (FVT), antisaccade (AS), prosaccade (PS), memory-guided saccade (MGS), smooth-pursuit eye movements (SPEMs), vergence, binocular rivalry (BR), and eye-blink rate (EBR). The focus areas of this review include (1) how methodological variation across task design, stimulus properties, and outcome measures may contribute to inconsistent findings in the literature; (2) which paradigms show potential(s) to be used for studying state markers, trait markers, or both in BD and their potential clinical utility for diagnosis, prognosis, and treatment monitoring.

## 2. Methods

### 2.1. Review Design

This methodology-focused narrative review used a structured literature-search and study-selection process. The Scale for the Assessment of Narrative Review Articles (SANRA) was used to self-appraise the completeness and transparency of narrative-review reporting [17]. The reporting of study identification and selection of studies was reported according to applicable items of the PRISMA Extension for Scoping Reviews (PRISMA-ScR) [18]. The review was not registered, and no protocol was published a priori.

### 2.2. Search Strategy and Eligibility Criteria

A literature search of PubMed and Scopus was performed from database inception through 10 August 2026. The search combined controlled vocabulary and free-text terms describing bipolar disorder with terms related to eye tracking, eye movements, and the prespecified ocular and oculomotor paradigms. The complete database-specific Boolean search strings, search fields, dates, and yields are provided in Supplementary Table S1. The reference lists of included studies and prior reviews [7,14,15] were searched manually to identify additional eligible studies.

Eligible studies were English-language original research articles involving adults (≥18 years) from at least one of the following groups: (1) individuals with a clinical diagnosis of BD (type I, type II, not otherwise specified, or bipolar spectrum disorder) established by recognized DSM or ICD criteria and structured diagnostic interviews; (2) individuals at familial high risk for or ultra-high risk for BD, identified using an explicitly defined clinical assessment. Studies were required to include at least one eye-tracking paradigm of interest, i.e., free-viewing tasks, antisaccades, prosaccades, memory-guided saccades, smooth-pursuit eye movements, vergence, binocular rivalry, or eye-blink rate, and to report at least one quantitative eye-tracking measure as an outcome.

Studies were excluded if they (1) focused on pediatric populations; (2) included psychiatric disorders other than BD (schizophrenia, major depressive disorder, obsessive-compulsive disorder, post-traumatic stress disorder, autism, or substance use disorders); (3) failed to report quantitative eye-tracking outcomes; or (4) were reviews, meta-analyses, case reports, editorials, or conference abstracts. Studies that were relevant to BD and eye tracking but did not provide extractable quantitative outcomes were excluded from the primary evidence synthesis and paradigm counts; however, they were mentioned briefly for contextual discussion when relevant.

### 2.3. Study Selection and Data Extraction

**Figure 1:**
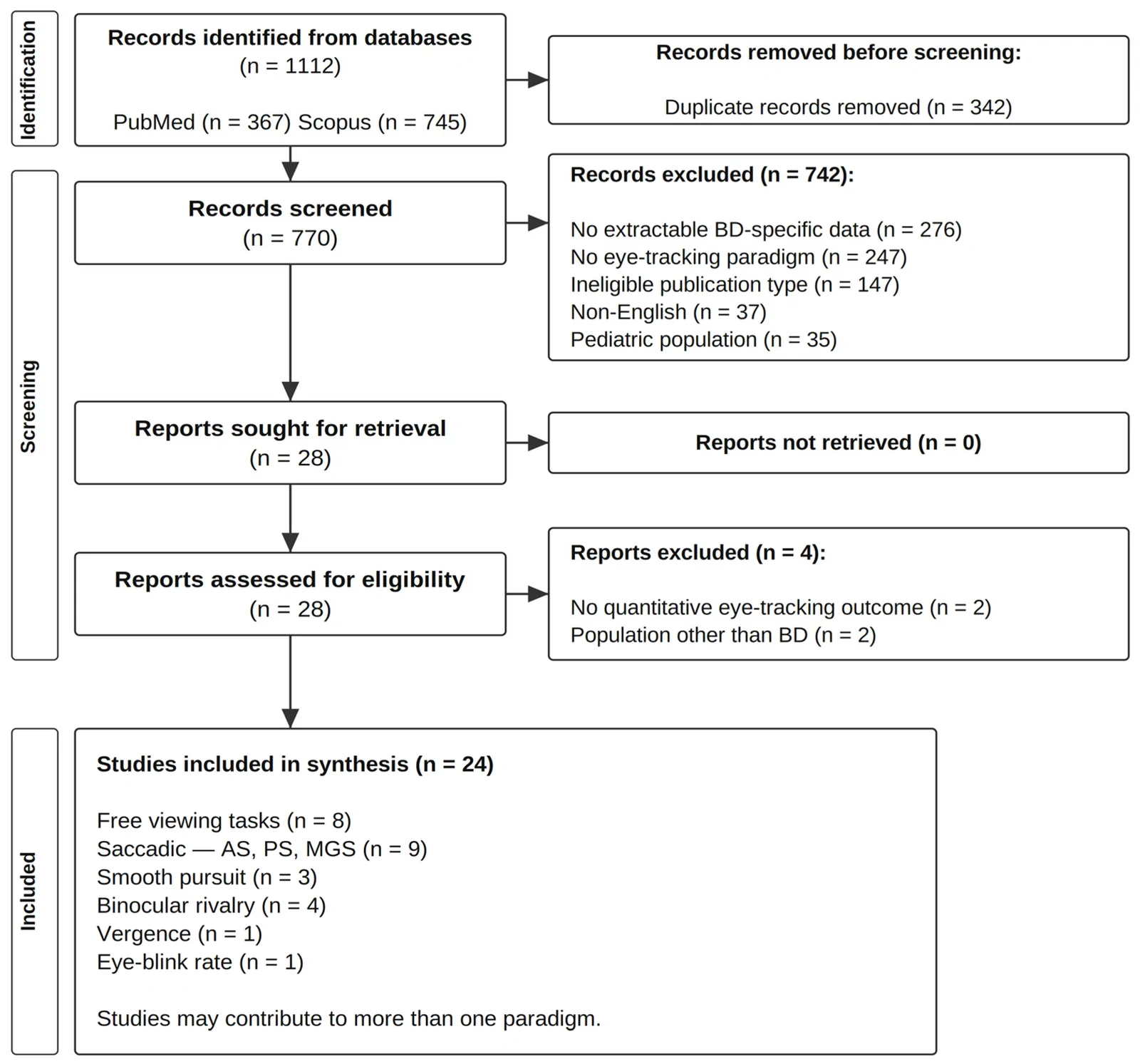
Study selection flowchart illustrating the identification, screening, eligibility, and inclusion of studies in the narrative review of eye-tracking paradigms in BD. Abbreviation: AS, antisaccade; BD, bipolar disorder; MGS, memory-guided saccade; PS, prosaccade.

Study selection was conducted independently by two authors (E.B.O. and B.K.) in three sequential stages: title screening, abstract screening, and full-text review. Disagreements at any stage were resolved through consensus discussion. At the full-text stage, two studies were excluded for not reporting quantitative eye-tracking outcomes [19,20]. The selection process is summarized in Figure 1. For each included study, the following data were extracted: study identifiers (author and year), sample characteristics (BD subtype, risk groups for BD, mood state, sample size), eye-tracking paradigm (task design, stimuli type, equipment used), and key outcome measures.

### 2.4. Categorization and Definitions of Eye-Tracking Paradigms

Since many variables can be measured using eye-tracking technology, each study used different designs to capture different domains of eye movements. To analyze eye-movement domains across studies, we categorized the literature into eight distinct paradigms: free-viewing task (FVT), antisaccade (AS), prosaccade (PS), memory-guided saccade (MGS), smooth-pursuit eye movements (SPEMs), vergence eye movements, binocular rivalry (BR), and eye-blink rate (EBR).

#### 2.4.1. Free-Viewing Task

FVT paradigms examine attentional bias and emotion regulation processes. Attentional bias eye-tracking paradigms have been used in research on autism, anxiety, major depressive disorder (MDD), and BD [21,22,23]. The primary outcome measures extracted from studies that used FVT included initial orienting assessed by first fixation location (% of total fixations), latency of first fixation (ms), first fixation duration (ms), and number of first fixations (%). Attentional engagement and sustained attention are commonly measured with gaze duration (ms), total fixation time (ms), and total number of fixations (Table S3).

#### 2.4.2. Saccadic Eye Movements

Antisaccade: AS tasks examine inhibitory control and executive function [24]. Key outcome metrics are error rate, i.e., the percentage of trials in which the first saccade is made toward the target rather than away from the target over all valid trials, and latency of correct antisaccades, i.e., the time (ms) from target appearance to initiate a correct antisaccade [25,26]. Additional measures used in BD research include the accuracy of first saccades (%), peak velocity of saccade (degrees/second), and position error, defined as the difference between gaze endpoint and required AS location in degrees. Other relevant metrics are corrected errors and uncorrected errors. Corrected errors are initial incorrect saccades followed by a correct antisaccade, and uncorrected errors are incorrect saccades toward the target not followed by a correction.

Prosaccade: PS tasks require minimal executive control and assess basic sensorimotor integration and reflexive orienting, with outcomes including error rate (%), latency (ms), and saccade gain. The saccade gain is a unitless ratio of saccade amplitude to target amplitude and reflects spatial accuracy [27]. This paradigm is commonly used as a control condition alongside antisaccade tasks [25,26].

Memory-Guided Saccade: MGS paradigms assess spatial working memory. When this paradigm is used, participants are instructed to fixate centrally while a peripheral target briefly appears. After a delay, participants are required to make a saccade to the remembered target location where the target appeared before [28]. Outcome measures include the number of correct saccades, i.e., saccades made to the remembered location of the target, the latency of correct saccades, i.e., the time (ms) taken from the disappearance of the peripheral target to initiate a correct saccade, amplitude of saccade as measured with degrees from central to target, peak velocity of saccades during eye movement as measured as degrees/seconds, and number of uncorrected errors. Additionally, in this paradigm, anticipatory and express saccades were measured. Anticipatory saccades are initiated before a target is presented, and express saccades are initiated immediately after a target is presented.

AS, PS, and MGS paradigms have been used in patients with MDD, BD, attention deficit hyperactivity disorder (ADHD), eating disorders, obsessive compulsive disorder (OCD), schizophrenia, and alcohol use disorders (AUDs) [29,30,31]. Since impulsivity, cognitive impairment, and executive dysfunction are common presentations of BD, especially during manic or depressive episodes [32,33], AS, PS, and MGS paradigms provide valuable metrics to assess cognitive control, inhibitory functioning, and spatial working memory in patients with BD.

#### 2.4.3. Binocular Rivalry

BR paradigms capture perceptual and attentional changes. Dichoptic training/therapy has been used in treating amblyopia (lazy eye) through forcing the brain to integrate different images presented to each eye simultaneously and to strengthen the weak eye. In BR research paradigms, different images, often vertical vs. horizontal gratings, are presented separately to each eye using dichoptic methods. When two eyes receive dissimilar, non-fusible images, the visual system does not integrate them into a single percept but engages interocular suppression, where one eye’s image dominates while the other is suppressed, which produces binocular rivalry [34,35]. Perception alternates over time: for one period, one eye’s image is dominant in awareness while the other is suppressed, then they swap. The main outcome measures were alternation rate (Hz), dominance phase duration (ms), accuracy rate (%), and response time (ms). BR paradigms have been applied in studies of BD, OCD, MDD, and schizophrenia [36,37]. Alterations in BR performance may reflect underlying deficits in visual perception, attention, motor speed, or memory, all of which are frequently impaired in BD.

#### 2.4.4. Smooth-Pursuit Eye Movements

SPEM paradigms evaluate predictive-tracking and motion-processing capabilities. The key outcome measures include position gain, i.e., a dimensionless ratio of eye position to target position, velocity gain, i.e., a dimensionless ratio of eye velocity to target velocity, pursuit initiation latency (ms), and initial eye acceleration [38]. SPEM paradigms have traditionally been considered an endophenotype for schizophrenia [39,40], but SPEM deficits are not specific to schizophrenia and have also been consistently reported in BD, particularly in those with psychotic features [41,42]. Functional imaging data also highlight abnormal cerebellar and frontoparietal activation during SPEM in BD, which might be related to ocular-motor dysfunction and underlying neurophysiological alterations [19].

#### 2.4.5. Vergence Eye Movements

Vergence paradigms investigate binocular coordination and depth perception. The key outcome measures are convergence and divergence errors (degrees), which assess the difference between the required vergence angle and the actual eye position. Larger errors indicate poorer accuracy of vergence control. Other outcome measures include vergence gain, defined as a ratio of the vergence response amplitude to the stimulus vergence demand, and number of saccadic intrusions (count) [43]. Although most ocular-motor research in BD has focused on horizontal plane tracking, vergence movements offer a distinct opportunity to examine visual-motor integration and depth processing. The cerebellar and frontoparietal regions, which exhibit both structural and functional irregularities in BD, play a role in supporting these eye movements [44]. Furthermore, BD shares several neurophysiological features with schizophrenia, a condition in which vergence impairments have been documented [45,46,47]. Given these overlaps, incorporating vergence paradigms into this review allows for a more comprehensive evaluation of oculomotor disturbances in BD.

#### 2.4.6. Eye-Blink Rate

EBR paradigms reflect dopaminergic tone, reward-driven behavior, and cognitive flexibility [48,49]. The outcome measure for this paradigm includes blink rate, which is defined as the number of blinks per minute (blinks/min). EBR has been used in research on Parkinson’s disease, schizophrenia, and ADHD, as a non-invasive biomarker of dopaminergic activity [50,51,52,53]. In BD, dopaminergic dysregulation, implicated reward sensitivity and motivational processes have been studied [54,55].

### 2.5. Analytical Approach

Given the marked heterogeneity across paradigms, stimulus types, mood-state sampling, and outcome measures, quantitative synthesis was not performed. Findings were synthesized narratively and organized by paradigm. Within each paradigm, studies were grouped by methodological features relevant to interpretation, including stimulus type (emotional vs. non-emotional), mood state at assessment (euthymic, depressive, manic, high-risk/prodromal), and outcome category.

Risk of bias was assessed independently by two reviewers (E.B.O. and B.K.). Observational group-comparison studies were evaluated using the revised JBI critical appraisal tool for analytical cross-sectional studies, whereas studies evaluating an intervention or within-participant treatment effect were evaluated using the revised JBI tool for quasi-experimental studies [56,57]. Each item was classified as “yes,” “no,” “unclear,” or “not applicable,” with disagreements resolved through discussion and consensus. Numerical summary scores were not calculated because the individual domains address different sources of bias and are not necessarily equally weighted. Studies were not excluded based on appraisal results; instead, the assessments informed interpretation of the evidence. Item-level judgments are presented in Supplementary Table S2.

## 3. Results

The database search identified 1112 records: 367 from PubMed and 745 from Scopus. After removal of 342 duplicates, 770 unique records underwent title-and-abstract screening. All 28 reports underwent full-text eligibility assessments, of which four were excluded, leaving 24 reports for inclusion in the narrative synthesis (Figure 1). The included studies comprised eight reports on free-viewing tasks, nine on saccadic paradigms, three on smooth-pursuit eye movements, four on binocular rivalry, and one report each on vergence and eye-blink rate. Because some reports investigated more than one paradigm, these categories were not mutually exclusive.

Across the 24 included studies, several used more than one eye-tracking paradigm. One study combined FVT with AS and PS [58], one combined AS with MGS [59], and one combined AS, PS, and SPEM [60]. In total, six studies reported both AS and PS results [58,60,61,62,63,64]. The paradigm types across the 24 studies comprised eight FVT, seven AS, eight PS, one MGS, four BR, three SPEM, one vergence, and one EBR.

### 3.1. Free-Viewing Task

Eight studies used FVT paradigms [58,65,66,67,68,69,70,71]. These studies varied substantially in the mood states of participants, definition of euthymia, stimulus type, trial structure, task instructions and outcome measures (Table 1). FVT studies in BD have employed diverse stimulus presentations. Some studies have employed side-by-side pairing of two images, using either facial expressions [58] or complex scenes from the International Affective Picture System (IAPS) [66,67]. IAPS scenes depict real-world situations. For example, happy images show people expressing positive affect or taking pleasure in activities, while threatening images depict hostile people or individuals facing serious threats or harm. Other studies used four images [65,68] or single images in a complex scene to quantify attention to specific emotional content within the scene through a detailed area-of-interest (AOI) analysis [69]. Kjærstad et al. (2020) extended this approach using highly emotional film clips instead of static emotional pictures to simulate more naturalistic, real-world attentional engagement over longer viewing periods [70].

Several studies incorporated additional methodological components beyond passive viewing. Peckham et al. (2016) combined passive viewing with self-report measures of dampening of positive affect and brooding (rumination on negative affect) [68]. Similarly, Broch-Due et al. (2018) incorporated explicit emotion regulation by instructing participants to “dampen” their response to negative images without specifying a particular strategy, contrasting this with a passive view condition [71]. García-Blanco et al. (2017) introduced a dual-block design with an inhibitory control component, requiring participants to selectively attend to either emotional or neutral images [67] (Table 1).

In studies using free-viewing emotional images, patients with BD were commonly compared with healthy controls (HCs). Some studies included patients in different mood states, including depressive, manic, and euthymic states, and others included only patients in a manic state or in a euthymic state. In studies including patients with different mood states, comparisons between mood groups were also conducted. The outcome measures were different in different studies. The most used outcome measures were total fixation time and total number of fixations. Other outcome measures included location of first fixation, latency of first fixation, number of first fixations, and duration of the first fixation (Table 1). To organize the synthesis, we grouped studies by paradigm design into four categories: (1) passive viewing of emotional images, (2) AOI-based passive viewing of emotional scenes, (3) passive viewing of dynamic video clips, and (4) free viewing with instructional conditions.

#### 3.1.1. Passive Viewing of Emotional Images

Four studies employed passive viewing of emotional images without task instructions [58,65,66,68]. These studies differed in several methodological aspects. Two studies used side-by-side pairings of two images for brief durations. One used the images from IAPS scenes for 3 s [66]. Another used facial expressions from the Chinese Facial Affective Picture System (CFAPS) for 5 s [58]. The other two used four-image arrays for extended viewing periods. One used IAPS scenes for 20 s [65], and another used facial expressions of the same actor from the Montreal Set of Facial Displays of Emotion (MSFDE) for 10 s [68]. Eye-tracking apparatus also varied, with studies using the SMI RED250 [65,66], SMI RED500 [58], or Tobii T120 [68] (Table 1).

Patients versus HCs: In one study, euthymic BD patients showed increased total fixation time and more total fixations on threatening images compared to HCs [65]. However, other studies found no difference between euthymic patients and HCs in total fixation time or number of fixations [66], location of first fixation [65,66,68], total fixation time or average fixation duration [68], mean glance duration [65], or latency and first-pass fixation duration [66] (Table 2).

Patients with mania showed increased total fixation time and more total fixations on threatening images compared to HCs [65]. Liu et al. (2019) found that patients with mania had a shorter total fixation time on sad and neutral images than HCs [58]. Manic patients also directed first fixations toward neutral images more frequently than HCs and showed longer first-fixation latency overall, with no group differences in duration of first fixation. García-Blanco et al. (2015) reported that manic patients and HCs did not differ in location, latency, first-pass fixation duration, or total fixation time and number [66]. Another study by García-Blanco et al. (2014) did not measure latency or duration of first fixation [65] (Table 2).

Depressed BD patients exhibited increased total fixation time and more total fixations on threatening images, alongside reduced attention to happy images compared to HCs [65]. In a different study from the same laboratory, depressed BD patients showed longer first-pass fixation durations than HCs overall, but latency did not differ between the groups [66]. The location of first fixation did not differ between depressed BD patients and HCs in either study (Table 2).

Mood states and valence interaction: Across all participants, emotional valence modulated attention. Threatening images elicited more and longer fixations than neutral images across all three BD mood states [65]. Happy images also received more total fixations than neutral images in manic patients, though this effect did not emerge for total fixation time [66]. In the study of Peckham et al. (2016), across all participants, happy faces received longer total fixation time than fearful or neutral faces, and sad faces received the shortest fixation time, but there were no significant differences between BD patients and HCs in any of these outcome measures [68]. Liu et al. found significant group differences for sad and neutral expressions but not for happy faces [58]. Both BD patients and HCs fixated first on happy images more frequently than on sad, threatening, or neutral images [65], and threatening images received longer first-pass fixation durations than neutral images across all participants [66] (Table 2). Temporal analyses further showed that attention to happy images increased linearly over the 20 s viewing period, threatening images showed both linear and quadratic trends (fixation time initially decreased then increased toward the end of the trial), and neutral/sad images remained stable. This pattern was similar for BD patients and HCs [65].

#### 3.1.2. AOI-Based Passive Viewing of Emotional Scenes

Purcell et al. (2018) employed a distinct methodological approach by using AOI-based analysis of a single emotional scene from IAPS that was recorded with an ASL Model 504 eye tracker [69]. Rather than presenting multiple images simultaneously, this paradigm presented one image at a time and examined whether participants preferentially fixated on predefined salient AOIs within each image compared with non-AOI regions. This paradigm shifted the focus from a binary choice between images to an exploration of attention within complex scenes. The study measured the percentage of time participants fixated on emotionally salient AOIs across the entire 5 s presentation, with separation into early (0.0–2.5 s) and late (2.51–5.0 s) phases (Table 1). The paradigm did not find significant differences in total fixation time on emotional AOIs between remitted BD patients and HCs, either for the overall presentation or for the early–late phase analysis. Neutral images received the highest overall fixation duration, followed by positive and then negative images (Table 2). Exploratory temporal analysis also found no significant group differences in how attention to emotional AOIs shifted between early and late phases. However, a post hoc analysis of non-emotional areas revealed that the BD group showed greater fixation on the non-emotional background of negative images in the late phase compared to HCs.

#### 3.1.3. Passive Viewing of Dynamic Video Clips

One study used dynamic video clips with themes of neutral, happy, sad, winning, racing, socially anxious and risk-taking for 90–110 s for each clip. The outcomes were attentional patterns of patients with BD in euthymia (remitted) and HCs established by analyzing AOI [70]. The AOI was defined as the whole computer screen, meaning the primary measure captured time spent viewing the screen versus looking away rather than within-image attention. Notably, this was the only study in the present review to include a mixed-subtype sample (60.5% BD-II, 39.5% BD-I). In addition to the eye-tracking measurements, the authors also assessed facial emotions of participants using the Affectiva Affdex algorithm (Table 1). Overall, there were no group differences in total fixation time and total number of fixations between euthymic BD patients and HCs. However, euthymic BD patients spent significantly less gaze time on the film clips than HCs. In clip-specific analysis, BD patients spent significantly less time viewing the neutral, happy, sad, winning, racing, and socially anxious clips compared to HCs. However, those differences between patients and HCs became non-significant after adjusting for subsyndromal symptoms and years of education (Table 2). The time spent on the risk-taking clip was not significantly different either. In contrast, euthymic BD patients exhibited more fearful facial expressions during film clips than HCs.

#### 3.1.4. Free Viewing with Instructional Conditions

Two studies extended passive viewing paradigms by incorporating explicit task instructions. García-Blanco et al. (2017) introduced a dual-block design with an inhibitory control component, in which participants were instructed to selectively attend to either emotional or neutral images [67]. Similarly, Broch-Due et al. incorporated explicit emotion regulation into a free-viewing paradigm [71]. In this paradigm, 48 unpleasant and 24 neutral pictures from the IAPS were presented individually for 4 s under three conditions of “View Neutral,” “View Negative,” and “Dampen Negative” without a specific instruction on strategies (Table 1). This approach is intended to resemble everyday strategies of a participant for emotional regulations.

Emotional reactivity was an outcome measured with commonly used free-viewing variables, including fixation time and number of fixations as well as facial expressions during each viewing (neutral versus negative) condition. The facial expressions were measured with the Affectiva Affdex algorithm. The Affdex system classifies seven emotions, including anger, sadness, disgust, fear, joy, surprise, and contempt, with a specific grading system. Emotional regulation was also an outcome that was measured as the degree of successful down-regulation of emotional responses when a participant viewed images under the different viewing instructions (view versus dampen negative). Emotional reactivity was examined using the condition of “view neutral” versus “view negative”. In contrast, emotional regulation was examined with “view negative” versus “dampen negative.”

In the study of García-Blanco et al. (2017), during free-viewing tasks with explicit instructions such as “attend-to-neutral” or “attend-to-emotion,” patients and HCs showed different attention patterns [67]. In the attend-to-neutral block, euthymic BD patients and HCs showed longer fixation time on neutral than emotional images. In the attend-to-emotion block, neutral images received less gaze duration than happy and threatening images across both groups. Within euthymic BD patients, during attend-to-emotion blocks, neutral images received less gaze time than happy and threatening images, whereas during attend-to-neutral blocks, neutral images received more gaze time than happy or threatening images. First-fixation latencies did not differ between euthymic BD patients and HCs. However, groups showed significantly different patterns of the percentage of initial fixations. Euthymic patients were only weakly influenced by emotional valence, with no significant differences between happy, threatening, and neutral images, whereas HCs showed increased attention to threatening compared to neutral images, with happy images falling in between. By contrast, both euthymic BD and HC groups showed a higher percentage of first fixations during attend-to-emotion than attend-to-neutral blocks (Table 2).

However, Broch-Due et al. found no difference in total fixation time and total fixation number between euthymic BD (*n* = 15) and HC (*n* = 16) groups during both emotional reactivity and emotional regulation [71]. Euthymic BD patients spent significantly less time gazing at both neutral and negative images than HCs during emotional reactivity and exhibited stronger facial expressions to neutral pictures. The BD patients also showed a lack of facial expressions to unpleasant pictures and more surprising facial expressions to both neutral and unpleasant pictures compared to HCs (Table 2).

The study of García-Blanco et al. (2017) [67] also found that manic and depressed BD patients exhibited significantly shorter total fixation times than both euthymic BD patients and HCs during attend-to-emotional blocks. In the attend-to-neutral task, manic BD patients spent more total fixation time on threatening and neutral images than on happy images, while depressed BD patients showed longer fixation time on neutral than on emotional images. In the attend-to-emotional task, depressed BD patients had shorter gaze duration for neutral than for happy and threatening images, while manic BD patients had longer gaze duration for happy images than for threatening or neutral images. Both manic and depressed BD showed significantly longer latency for the first fixation compared to both HCs and euthymic BD patients. Manic BD patients had a higher percentage of first fixation on happy images than threatening and neutral images regardless of task instructions. Task instructions significantly modulated the percentage of initial fixations on target images in HC, euthymic BD, and manic BD groups but not in the depressed group (Table 2).

### 3.2. Saccadic Eye Movements

In addition to attentional patterns during the free-view tasks, the speed or error of looking away from an emotional target (AS) and the speed of looking toward an emotional target (PS) were assessed in BD patients in different mood states as well as HCs. Nine reports examined saccadic paradigms. Seven studies used AS paradigms. Six of them studied combined AS and PS tasks [58,60,61,62,63,64], one study combined these with SPEM, and one study with MGS. Two studies employed only PS tasks [20,72] (Table 3).

#### 3.2.1. Antisaccade

Antisaccade with emotional images: Among the seven studies including AS measures, two studies employed emotional facial stimuli to investigate how mood state influences inhibitory control [58,61]. The emotional images were from the FACES database [73] for García-Blanco et al. (2013) [61] and from CFAPs for Liu et al. [58]. The studies were conducted with SMI RED250 [61] and SMI RED500 [58] eye trackers (Table 3).

In the study including patients at different mood states [61], patients in manic or depressive states showed significantly more AS errors than HCs. Patients in manic states made significantly more errors with happy faces than neutral or sad faces. Depressed patients showed a non-significant trend toward more errors with sad than with neutral faces. In contrast, there were no significant differences between the euthymic BD and HC groups in overall AS error rate, at 13.3% versus 19.3%, respectively (Table 4). In terms of AS latencies, patients at any state (manic, depressive, euthymic) had significantly longer AS latencies compared to HCs. In addition, this study also found significant positive correlations between mood congruency error scores and symptom severity, including sad–neutral error scores with BDI-II (Beck Depression Inventory II) (r = 0.389, *p* = 0.001) and happy–neutral error scores with YMRS (Young Mania Rating Scale) (r = 0.275, *p* = 0.020).

However, in the study that included only manic patients [58], neither AS error rate (*p* = 0.065) nor AS latency (*p* = 0.063) differed significantly between manic BD and HC groups, though both values were at the threshold of conventional significance (Table 4 and Table 5). This pattern in the study of Liu et al. [58] stands in contrast to García-Blanco et al. (2013) [61], where AS error rate was significantly elevated in manic BD (47.7% versus 19.3%, *p* < 0.001). The discrepancy between these two BD-I manic samples may reflect methodological differences, including stimulus exposure duration, i.e., 1600 ms in the study of García-Blanco et al. (2013) [61] versus 1000 ms in the study of Liu et al., stimulus database (FACES versus CFAPs), and/or fixation timing (fixed 1600 ms versus random within range of 1000–1500 ms) (Table 3).

Antisaccade with non-emotional stimuli: Three studies used non-emotional stimuli to assess inhibitory control independently of emotional salience [59,60,63]. Dyer et al. (2026) [63] examined BD-I and HC groups using central fixation crosses and peripheral circle targets. Ekin et al. (2023) [59] and Ritish et al. (2024) [60] focused on early or prodromal stages of BD and used non-emotional visual targets on a black background. Ekin et al. (2023) [59] used a gray plus sign and gray circles, while Ritish et al. (2024) [60] used targets of unspecified shape at ±6° and ±12° lateral positions. All three employed high-precision Eye Link 1000 systems (Table 3). Ritish et al. investigated remitted patients within 6 months after first-episode mania (FEM), individuals at familial high risk for BD (HR-BD) (siblings or offspring of patients with BD) and HCs [60].

Dyer et al. (2026) [63] found no significant overall BD–HC differences in antisaccade error rate (*p* = 0.088) or latency (*p* = 0.068) (Table 4 and Table 5). Within the BD group, longer latency was significantly associated with poorer spatial working memory after correction for multiple comparisons. Exploratory subgroup analyses indicated higher error rates or longer latencies among BD participants with poorer domain-specific cognitive performance, whereas stratification by global cognitive performance did not identify significant differences [63].

FEM patients exhibited significantly higher AS error rate compared to HCs, 81.6% versus 50.6%, respectively (*p* = 0.01). However, HR-BD individuals showed an AS error rate of 64.3%, but this difference was not statistically significant compared to FEM or HC groups (Table 4). This study also reported position errors, in which FEM patients exhibited significantly more position errors compared to HCs (*p* < 0.01), while HR-BD individuals again showed non-statistically significant impairment compared to FEM and HC groups. However, there were no significant group differences in latency or peak velocity [60].

As a part of an antisaccade study in individuals with ultra-high risk for bipolar disorder (UHR-BD), Ekin et al. [59] used three symptoms proposed by Bechdolf et al. to identify the study subjects. The three symptoms include brief-intermittent manic syndrome, attenuated manic syndrome, and genetic risk and deterioration syndrome [74]. The AS task comprised 64 test trials (after four practice trials) using a gap paradigm: 1000 ms central fixation → 200 ms gap → target (gray circle) displayed up to 1000 ms (Table 3). During the AS experiment, study subjects were instructed that when a dot (gray circle) appeared in one of the squares on the black screen, they should look at the center of the square on the opposite side of the dot. Compared to HCs, subjects with UHR-BD showed significantly fewer correct saccades (*p* = 0.02), and significantly more anticipatory (*p* = 0.009) and express saccades (*p* = 0.040). For the AS task, UHR-BD subjects did not differ significantly from HCs in latency (*p* = 0.142), corrected errors (*p* = 0.197), or peak velocity (*p* = 0.873). However, amplitude of AS showed a borderline effect (*p* = 0.050), and uncorrected errors of AS showed a trend toward higher value in UHR-BD subjects (*p* = 0.097).

Antisaccade with a mixed saccadic task paradigm: Two studies used antisaccade paradigms with additional cognitive demands through mixed trial types [64] or emotional contexts with attentional modulation [62]. Beynel et al. (2014) [64] employed a SPAN (Saccade: Pro, Anti, No-saccade) task, which differs from the traditional AS paradigm. SPAN is a mixed eye-tracking paradigm that uses PS (look at target), AS (look opposite target), and no-saccade (NS) trials (fixate centrally) to increase the cognitive load when assessing executive functions (Table 3).

In a pilot study (*n* = 12) of intermittent theta burst stimulation (iTBS) in drug-resistant bipolar depression, Beynel et al. [64] used a SPAN task paradigm to assess AS performance immediately before and after active versus sham treatment and examined if changes in AS performance were associated with subsequent mood improvement. They found that patients in a depressive state showed significantly more inhibition (AS) errors than HCs, 26.9% versus 13.8%, respectively, but showed no significant difference in PS, NS, and AS latencies in AS performances. They also found a significant positive correlation between pre–post AS error and improvement in Montgomery–Åsberg Depression Rating Scale (MADRS) scores (r = 0.65; *p* = 0.02). Following iTBS treatment, the active group (*n* = 5) showed a significant reduction in AS error rate, from 23.2% to 14.9% (*p* = 0.01), while the sham group (*n* = 7) showed no significant change, from 25.1% to 24.8%.

Another study introduced a dual design incorporating emotional (hot) and non-emotional (cold) stimuli to both step and gap conditions [62] (Table 3). In the cold AS task, a central fixation cross occurred for either 1000 or 1500 ms, followed by a circle target appearing peripherally either immediately (step trials) or after a 200 ms blank interval (gap trials). In the hot AS task, peripheral targets were positive (joyful) or negative (sad, fearful) emotional images from the IAPS, presented under similar “step” and “gap” conditions. In their analyses of “direct” comparison of patients with HCs, the authors used “cold” stimulus as neutral stimulus, “hot” stimulus of joyful images as positive stimulus, and sad/fearful images as negative stimulus. Furthermore, the authors created an emotionally charged overall variable for comparison (Table 4 and Table 5). In addition to this commonly used approach for comparison, the authors also used difference in error rates and latencies to different stimuli within patient and HC groups to create four variables, i.e., the contrast of “neutral-emotional,” “neutral-positive,” “neutral-negative,” and “negative-positive” for comparison (Table 4 and Table 5). Both types of comparisons were analyzed under “step,” “gap,” and “step + gap” conditions. The “step” condition is like other commonly used designs for antisaccade tasks. We present the results of error rates and latencies under “step” conditions (Table 4 and Table 5).

Using this step-gap design, the authors found no significant differences in AS error rates or AS latency overall under both conditions (Table 4 and Table 5). Both BD and HC groups had higher error rates for neutral than emotional stimuli and performed worse in the “step” condition across all stimulus types. In the BD group, AS latencies were longer for neutral than emotional stimuli overall, driven mainly by slower responses to neutral than positive stimuli, and there was also a slight negativity bias, with slower responses to negative than positive stimuli, particularly in the combined and step-trial analyses.

The within-group contrast between stimuli comparisons of “neutral-emotional,” “neutral-positive,” “neutral-negative,” and “negative-positive” also did not find significant differences between patients and HCs in error rates under “step + gap,” “step,” and “gap” conditions. However, the latency in negative-positive contrast was significantly longer in euthymic BD patients than in HCs under “step” (Table 5) and “step-gap” conditions [62].

#### 3.2.2. Prosaccade

Six studies discussed in the AS section also included prosaccade (PS) tasks [58,61,62,63,64]. The study of Dyer et al. included PS performance to verify that patients did not have a general saccadic deficit and establish a baseline for task performance, but PS outcomes were not reported in the primary analysis [62]. Two additional studies employed PS tasks exclusively, using electrooculography (EOG) with light emitting diode (LED) arrays rather than video-based eye tracking [20,72] (Table 3).

In the study including patients across mood states [61], depressed and manic patients exhibited significantly higher PS error rates than both HCs and euthymic patients, with no difference between euthymic BD and HC groups. In the study including patients in manic state [58], there was no difference in PS accuracy rate between manic patients and HCs. Regarding PS latency, patients in depressive and manic episodes showed longer PS latencies than HCs, whereas euthymic BD patients did not differ significantly from HCs [61]. Across all participants (BD and HC), PS latencies were slower for sad than for happy faces, with a non-significant trend for sad versus neutral [61]. Similarly, Liu et al. reported that manic patients had significantly longer PS latencies than HCs across all emotions [58]. In contrast, Velasques et al. (2013) using EOG and a predictable-target LED paradigm reported an opposite pattern [72], i.e., manic patients exhibited significantly faster PS latencies than both depressed BD patients and HCs, while depressed BD patients did not differ from HCs. In HR-BD subjects and patients after the first mania episode (FEM), Ritish et al. [60] found no significant differences in PS error, latency, peak velocity, or position error between FEM, HR-BD, and HC groups.

#### 3.2.3. Memory-Guided Saccade

Ekin et al. used the MGS paradigm to examine visuospatial perception and working memory in UHR-BD versus HC groups as part of the same study reporting AS findings above [59]. The MGS paradigm structure was 1000 ms fixation (red plus) → 200 ms cue (red target at 5° eccentricity) → 2000 ms delay → up to 1000 ms response (participant saccades to remembered target location) → 800 ms feedback if timed out. Tasks included 44 test trials plus four practice trials (Table 3). Compared to HCs, individuals with UHR-BD showed fewer correct saccades (*p* = 0.031), more uncorrected errors (*p* = 0.009) and more anticipatory saccades during both the cue and delay screens. Express saccades were significantly more frequent in UHR-BD only during the cue screen and not during the delay screen. However, no significant differences were observed for other MGS parameters, including saccade latency, amplitude, and peak velocity.

### 3.3. Binocular Rivalry

Four studies examining binocular rivalry (BR) in BD employed broadly similar presentation of stimuli (gratings) to the eyes and the same method of capturing responses [37,75,76,77]. However, study paradigms were different, and patient populations were diverse. Pettigrew and Miller (1998) included euthymic patients with BD (*n* = 18) and HCs (*n* = 49) [37]. In the study of Nagamine et al. (2009), euthymic patients with BD-I (*n* = 11) and BD-II disorder (*n* = 17) and HCs (*n* = 25) were included [75]. Vierck et al. (2013) included patients with BD-I (*n* = 71), BD-II (*n* = 22), bipolar disorder not otherwise specified (*n* = 3), and HCs (*n* = 24) [76]. Mood states in this cohort were euthymic, depressive, manic/hypomanic, and mixed, but analyses were primarily reported by diagnostic subgroup rather than mood state. In contrast, Law et al. (2017) included clinically stable patients with BD (*n* = 20) and HCs (*n* = 20) [77].

All four studies presented a vertical moving grating to one eye and a horizontal grating to the other, with a trial duration of 100 s per trial to induce spontaneous perceptual alterations between the two stimuli, but the number of trials and time of break between trials and blocks were different. All trials included a practice/stabilization trial that was excluded from analysis. Pettigrew and Miller excluded the first block as a training block, whereas Nagamine et al. additionally used a catch trial to familiarize participants with the task [37,75].

Stimulus details varied across studies. Pettigrew and Miller used drifting square-wave gratings presented using liquid-crystal shutters [37]. Nagamine et al. used red-gray vertical gratings and green-gray horizontal gratings [75]. The vertical gratings were presented to one eye and moved rightwards, and the horizontal gratings were presented to the other and moved vertically. Vierck et al. used drifting black-and-white striped gratings [76]. In contrast, Law et al. used two grating schemes, including green gratings and red/blue gratings. In the green grating scheme, the vertical grating moved rightwards, and the horizontal gratings moved downwards. In the red/blue grating scheme, the red vertical gratings moved rightwards, and the horizontal grating moved downwards [77]. In addition, the association of BR with cognitive functions was assessed in one study [76], and incorporation of other oculomotor paradigm like saccades and smooth tracking with BR was in another study [77].

The outcome measures also varied. Mean phase duration, i.e., the average time of the dominance period that excluded the period of fused or competitive perception was used as a primary outcome in one study [75]. Alternation rate was used in three studies [37,76,77]. Alternation rate (Hz) was calculated as the number of perceptual switches/alternations divided by the total duration of rivalry viewing, which excluded mixed/mosaic/unusual percepts and erroneous responses. In addition, Nagamine et al. also reported mean accuracy rate and response time of the catch trial to evaluate task performance [75].

In terms of results, Nagamine et al. found euthymic BD-I patients had significantly longer mean phase duration than both euthymic BD-II patients and HCs. However, HCs showed higher accuracy and shorter response times than both BD groups [75]. BD-I patients had significantly slower BR alternation rates than HCs, which was replicated by three other studies [37,76,77]. However, Vierck et al. did not find a significant difference in alternation rate between BD-I and broader bipolar-spectrum groups [76]. Law et al. further reported that BR alternation rate was not explained by other primary eye-movement measures, supporting the interpretation that BR captures a distinct perceptual process [77]. Thus, despite methodological and clinical differences, all four studies found evidence of slower rivalry in at least one BD group relative to HCs.

### 3.4. Smooth-Pursuit Eye Movements

Three studies investigated SPEM in BD [60,78,79]. Brakemeier et al. (2020) and Ritish et al. (2024) employed step-ramp paradigms [60,79], whereas Holzman et al. (1991) used sinusoidal and triangular target motion to examine changes in SPEM following lithium initiation [78]. Step-ramp paradigms are standard experimental setups for studying smooth pursuit. In this paradigm, a target suddenly moves to a new location (step) and then immediately starts moving at a constant velocity (ramp) [80]. This paradigm can test the ability of the brain to integrate positional and motion cues to initiate and maintain accurate eye movement, which can reveal neural delay and adaptation. Analyzing the velocity, acceleration, and saccades of eye movements can help understand the response of the ocular-motor system to predictable but complex stimuli and separate initial open-loop responses from later closed-loop corrections.

Holzman et al. (1991) evaluated 11 hospitalized patients with manic BD before and during lithium treatment and 13 HCs, assessed twice [78]. Using sinusoidal and triangular target motion, they found that the number of patients with normal pursuit decreased from 9 of 11 before lithium to 4 of 11 during treatment, with significant deterioration in qualitative pursuit ratings (group-by-time interaction, *p* = 0.02). Lithium was also associated with increased saccadic intrusions, while pursuit gain decreased in 8 of 11 patients without a significant group-by-time interaction. These changes occurred despite clinical improvement.

Brakemeier et al. (2020) examined specific sensorimotor components of pursuit initiation and maintenance in bipolar patients with psychotic features (BPwP), bipolar patients without psychotic features (BPwoP), and HCs [79]. BPwoP did not differ from either BPwP or HCs in initial eye acceleration, but they performed significantly better than BPwP on early (*p* = 0.004) and predictive (*p* = 0.017) pursuit maintenance measures. However, both measures did not differ from HCs. Compared to HCs, BPwP did poorly on initial eye acceleration and on early and predictive pursuit maintenance (all *p* < 0.01). No group differences were observed in pursuit latency. In contrast to the three pursuit measures, BPwP and BPwoP were both impaired in general neurocognitive assessments in relation to HCs (both *p* < 0.001), without a significant difference between the two bipolar patient groups [80].

Ritish et al. [60] examined SPEM in patients with remitted BD-I after the FEM, HR-BD subjects, and HCs using a moving, red-colored circle in 10 fast and 10 slow trials. In each trial, the red-colored circle moved from the center position to an unpredictable initial target (step) that was opposite to the direction of a subsequent target motion (ramp). Each trial had six-and-a-half ramps. The outcome measures were position gain and velocity during slow and fast SPEM trials. In patients with FEM, significant impairments in both position and velocity gain during both slow and fast trials compared to HCs were observed. In individuals with HR-BD, the impairment of SPEM performance was less severe than patients with FEM but more severe than HCs. However, the differences between FEM vs. HR-BD or HC vs. HR-BD groups were not statistically significant.

### 3.5. Vergence Eye Movements

The coordinated inward (convergence) or outward (divergence) rotation of the eyes to maintain binocular fusion when viewing distance changes is considered a marker of basic binocular motor control. Near point of convergence (NPC) is a basic parameter of the visual system and represents the amplitude of convergence and is commonly used as a measure for the integrity of the visual system. Chrobak et al. (2020) examined vergence eye movement in euthymic BD patients versus HCs using a binocular infrared eye-tracking system [81]. There was no significant difference between euthymic BD patients and HCs in NPC break and recovery points. However, compared to HCs, euthymic BD patients exhibited significantly increased convergence errors, a higher number of saccadic intrusions during convergence tracking, and more saccadic intrusions during divergence.

### 3.6. Eye-Blink Rate

Eye-blink rate (EBR) and its associations with self-reported ambition, confidence, and reward sensitivity are considered as a non-invasive marker of dopaminergic activity in BD. Only one study has directly investigated EBR in BD [82]. This study was to test whether phasic changes in EBR could provide an index of reward pursuit in patients with BD-I. Thirty-one adults with BD-I and twenty-eight HCs completed a task that involved effort toward monetary reward. Blink rates were recorded at baseline, reward anticipation phase, and post reward/reward receipt phase.

The baseline EBR was calculated by recording the number of eye-blinks in 5 min of looking at a fixation cross in the center of an eye-tracking computer and then dividing by five. The reward task was to solve as many anagrams as possible in 5 min to determine if a participant won money or not. EBR during reward anticipation was calculated by counting eye blinks in the 60 s immediately before the reward task started, and post-reward EBR was calculated by the eye blinks of the 60 s immediately after the reward task ended.

To complete a reward task, an on-screen cue stating whether the next trial was easy, medium, or difficult was provided to participants in a random manner immediately before each trial. No feedback to participants on winning was given until the end of the 5 min task. To enhance reward anticipation, the anagram task was preceded by a “countdown” of 60 s from 60 to 1, with “Get Ready!” on the screen (phase of reward anticipation). To further enhance anticipation, a clip of upbeat music during the count-down minute was presented to participants. The same music was played again immediately upon completing the 5 min task for 60 s while participants were viewing their winnings on screen, to enhance the positive effects of winning money on the task (phase of post reward/reward receipt). The “countdown” phase only occurred once, immediately before the start of the 5 min anagram block. Before completing reward task, patients with BD-I also completed clinician-rated depression and mania scales and self-report measures relating to reward and ambition.

There were no significant differences in EBR between BD-I and HC groups across task phases with levels of anagram difficulties. However, EBR changed significantly over time across both groups. EBR marginally increased from baseline to reward anticipation (*p* = 0.08) and significantly increased from reward anticipation to reward receipt (*p* = 0.04) in both groups. Within the BD group, after controlling baseline EBR, EBR during reward anticipation was positively correlated with ambition and goal setting (r = 0.43) and confidence (r = 0.41), and EBR during the post-reward phase was associated with higher scores on a reward-triggered mania scale (r = 0.41).

## 4. Discussion

In this methodology-focused narrative review, we synthesized evidence across eight eye-movement paradigms applied in BD. Eye-tracking technology in studying BD has advanced, especially in the last decade, particularly through image-viewing or attentional-bias paradigms [22]. FVT, also known as attentional bias paradigms, are the most-used eye-tracking approach in BD research, although the study designs were different among the different studies (Table 1). More complex paradigms, including viewing images with instructions [67,71] or antisaccade tasks [58,61], have been explored in bipolar research. AS and PS are the second most-used paradigms (Table 3). Across paradigms, the evidence suggests that eye tracking can capture abnormalities in affective-attentional processing, inhibitory control, perceptual dynamics, sensorimotor coordination, and reward-related behavior in BD. However, no paradigm is currently supported as a tool to identify clinical biomarkers for diagnosis, prognosis, or treatment monitoring in BD.

So far, no reliable FVT-based biomarker has been identified that consistently distinguishes patients with BD from HCs. A recent meta-analysis found that patients with BD showed longer first fixation latency, fewer fixations, shorter gaze duration, and lower saccadic peak velocity and amplitude compared to HCs [15]. However, several pooled estimates were based on only two or three studies, and pooling can identify an average group effect despite variability among individual studies. The present review complements these findings by examining study-level methodological and clinical heterogeneity while recognizing that pooled BD–HC differences alone do not establish individual-level biomarker validity.

Inconsistent findings among the studies could be due to differences in symptom severity, definitions of euthymia or remission, stimulus design, task demands, and outcome selection (Table 1 and Table 2). In particular, broader remission criteria in Broch-Due et al. and Kjærstad et al. may allow clinically relevant residual symptoms, and gaze findings in both studies were attenuated after adjustment for subsyndromal mood symptoms [70,71]. Their findings therefore cannot necessarily be attributed to state-independent abnormalities. Stimulus type also ranged from emotional images [58,65,66,68] and emotional scenes [69] to dynamic film clips [70]. Moreover, passive viewing tasks primarily index bottom-up attentional bias, whereas instructed or condition-based paradigms (e.g., attend-to-emotion vs. attend-to-neutral blocks) [67,71] recruit top-down regulatory control, making results not directly comparable across these designs [83,84]. Furthermore, variations in mood-state sampling and the specific eye-tracking metrics reported hinder cross-study comparisons and the identification of biomarker candidates (Table 1 and Table 2).

It remains unclear whether any single FVT design has a clear methodological advantage over the others. The heterogeneity in the designs reveals that each approach probes a distinct aspect of attentional processing. The choice of design has been and will be guided by the research question, since no single design holds a universal advantage. The value of using interference or condition to view image paradigms remains unclear, although similar results were found compared to paradigms without inference or condition. Any additional modifications to free-viewing image paradigms will add complexity for emotional and cognitive processing. Similarly, the value of using non-emotional stimuli or video clips to study emotional and cognitive process in patients with BD is unclear, although similar findings to studies using emotional images were reported in a systematic review and meta-analysis [15]. Clearly, the field needs standardized design elements, such as definition of each mood state, unified stimulus presentation, outcome metrics, and analytic methodologies, through a consensus of the experts in the field. In doing so, FVT-based eye-tracking studies may find consistent results between patients with BD and HCs or patients with psychiatric disorders, including MDD and schizophrenia, to determine if FVT-based eye-tracking paradigms are a valid tool to identify biomarkers for the diagnosis and treatment of BD.

The field may also benefit from refining the goals of FVT research in BD. Most available studies compared BD only with HCs, which is useful for characterizing abnormalities in emotional-attentional and cognitive-affective processing but less informative for determining whether these abnormalities are specific to BD or shared across psychiatric disorders. Including manic and depressive states is important for evaluating state-related effects, but current FVT evidence does not yet identify a stable mood-state-specific biomarker (Table 2). Current evidence does not indicate that manic-state inclusion has yielded a stable or diagnostic specific biomarker, and the meta-analytic findings suggest that patients in mania show broadly similar emotional-cognitive alterations to those observed in euthymic or depressive states relative to HCs [85]. Even if the results during a manic state are confirmed as a state biomarker for patients with BD, they are still less useful in diagnosing and treating patients with BD compared to the results during a depressive episode, which can be potentially used in differentiating bipolar depression from MDD.

Similar to FVT findings, AS findings in BD relative to HCs are inconsistent, although there were fewer AS studies than attentional bias studies. These discrepancies might be explained by methodological differences in mood state at assessment, stimulus types, task structure and outcome measures (Table 3). Studies using emotional face stimuli suggest a possible dissociation in which AS error rate may be more sensitive to current symptom (manic or depression) state (Table 4), whereas AS latency may remain elevated across mood states, including euthymia (Table 5). However, this pattern is not consistently replicated across studies and should therefore be interpreted cautiously. The contrast between the studies of García-Blanco et al. (2013) [61] and Liu et al. [58], despite broadly comparable manic BD-I samples, illustrates how differences in stimulus exposure duration, stimulus set, and fixation timing may attenuate or eliminate apparent group effects. By contrast, Dyer et al. (2025) [62] found no significant differences in AS error or latency in euthymic BD, although both BD and HC groups showed similar within-group effects of stimulus valence and step-gap modulation (Table 4 and Table 5). Dyer et al. (2026) also found no significant overall BD–HC differences in AS error rate or latency, although exploratory analyses suggested that poorer performance may be concentrated among BD participants with deficits in specific cognitive domains [63].

The study of Dyer et al. (2025) [62] is worthy for noting that using “step” and “gap” design can increase the complexity of cognitive processes. The insignificant differences between patients with BD in a euthymic state and HCs under multiple emotional stimuli suggest that the levels of cognitive inhibition may not be a trait biomarker for patients with BD. However, it is worth using the same design to examine the cognitive inhibition of patients with BD in a depression or manic state. Previously, we found that patients with BD in a euthymic state had similar levels of function and quality of life [86,87], suggesting that when patients with BD are in euthymia, they may have fewer differences relative to HCs compared to when they are in a manic or depressive state.

The aforementioned findings suggest that emotional salience may be an important methodological factor in revealing inhibitory-control abnormalities in BD. The current literature also suggests that AS error and latency may capture partially different aspects of inhibitory control. Error rate may more directly reflect failed inhibitory control, whereas latency may reflect processing speed and response preparation [88]. Non-emotional AS paradigms related to reward and impulsivity have been used in studies of patients with substance use disorders [89,90]. The utility of such paradigms should be explored in BD research (www.clinicaltrials.gov, NCT03829787).

As a potential predictive biomarker for BD in HR-BD or UHR-BD subjects [59,60], the results of AS were not significantly different from HCs (Table 4 and Table 5). However, abnormalities in anticipatory and express saccades in UHR-BD subjects were observed [59], which raises the possibility that abnormalities in saccade initiation and preparatory control can be considered as an earlier presentation for BD in HR-BD and UHR-BD subjects.

The PS findings are similarly inconsistent, and methodological context is essential for interpretation. Longer PS latencies in symptomatic manic and depressive states may reflect generalized psychomotor slowing or altered sensorimotor processing during mood episodes. However, the opposite pattern, namely faster PS latencies in mania, reported by Velasques et al. [72], suggests that paradigm structure may substantially shape the observed effect. The study used EOG and a predictable LED-based target paradigm, both of which may facilitate anticipatory responding and differ substantially from video-based eye-tracking paradigms using less-predictable targets. Accordingly, the faster latency finding in mania should be interpreted cautiously and not assumed to reflect a robust disease-specific effect. In contrast, the stimulus-valence effect reported by García-Blanco et al. (2013) [61], in which sad faces elicited slower PS latencies than happy faces across participants, appears to reflect a more general stimulus-driven effect rather than a BD-specific abnormality. This is consistent with the theoretical dissociation between top-down (AS) and bottom-up (PS) attentional processes [26], in which voluntary inhibitory control is susceptible to individual affective state, while reflexive orienting is more influenced by stimulus properties.

The single available MGS study provided preliminary evidence that abnormalities seen in AS performance may extend to working-memory–dependent saccadic tasks in UHR-BD subjects [59]. However, because this evidence comes from a single small cross-sectional sample, the findings require replication before they can be considered potential markers of bipolar risk. Adding memory components to saccade and antisaccade tasks is likely to increase the executive function load so that a combination of MGS and AS tasks may provide more information than either task alone.

The four available BR studies provided relatively consistent evidence that BR alternation was slower in BD than in HC groups, even though grating configurations and task implementations were somewhat different [37,75,76,77]. The consistent results suggest that BR might be one of the more robust perceptual abnormalities identified in the BD eye-movement literature. However, slowed BR alternation is not specific to BD. Ye et al. (2019) demonstrated similarly reduced rivalry rates in BD, MDD, OCD, schizophrenia, and first-degree relatives of schizophrenia patients [36], suggesting that BR slowing may reflect a transdiagnostic alteration in perceptual dynamics rather than a disorder-specific marker. In addition, Jia et al. (2015) showed that in unipolar depression, BR slowing was most pronounced during depressive episodes and partially normalized during remission [91], suggesting that a slowed BR might be a state-related component in MDD. In the current review, patients in different mood states were included in BR paradigms, but the results of BR alterations were consistent, suggesting that the slowed BR in BD may be considered as a candidate for a trait marker of BD. However, the available studies were not designed to test this directly. Future studies should move beyond alternation rate alone and examine multiple BR parameters, such as dominance-duration asymmetry and variability of perceptual switching. Study subjects should include BD patients with different mood states and different psychiatric groups to determine whether BR can help to distinguish bipolar depression from unipolar depression and/or other psychiatric disorders and to clarify state versus trait significance.

SPEM deficits appear to be more closely associated with psychosis vulnerability than BD itself. The finding of SPEM deficits primarily in bipolar patients with psychotic features [79] is consistent with the broader psychosis literature, which reports that SPEM abnormalities may represent a shared neurophysiological alteration across schizophrenia spectrum disorder and BD with psychotic disorder rather than a BD-specific marker [41,45]. However, Ritish et al. found impaired position and velocity gains in patients following first-episode mania [60], while Holzman et al. demonstrated that lithium treatment itself can impair pursuit performance [78]. These findings indicate that illness stage, psychosis history, and medication exposure may all influence SPEM outcomes. Overall, the evidence of SPEM in BD remains limited, and the available findings are insufficient to conclude that SPEM is specifically a trait marker or that it reliably distinguishes BD from schizophrenia.

The clinical relevance of vergence eye movements is unclear, although patients with BD showed significant vergence abnormalities, including increased convergence errors and saccadic intrusions, during both convergence and divergence tracking compared to HCs [81]. These findings indicate that vergence paradigms may capture aspects of visual-motor dysfunction that are not reflected by more basic measures, such as NPC alone.

The results of EBR in BD are interesting, although there were no significant differences in EBR between BD and HC groups across task phases with different levels of task difficulties [82]. A trend significance of EBR from baseline to reward anticipation and significant difference from reward anticipation to reward receipt in both groups suggest that EBR may not be useful to study the difference in this measure between BD patients and HCs in different phases of the reward process. However, the findings within the BD group, i.e., phasic EBR measures were associated with ambition, confidence, and reward-triggered mania scores, suggest that using EBR may identify a subgroup of patients with BD who are likely to be involved with high-reward activities. Clearly, whether EBR has value in identifying clinically meaningful bipolar subgroups, including those with altered reward sensitivity, is worthy of further exploration.

Medication exposure represents an important source of heterogeneity. Benzodiazepines, antipsychotics, anticholinergics, and some anticonvulsants can impair saccadic or smooth-pursuit performance [92]. A recent meta-analysis confirmed a large benzodiazepine-related reduction in saccadic peak velocity and increase in latency [93]. Medication may also obscure EBR differences; a meta-analysis found elevated EBR in unmedicated but not medicated psychiatric samples [51]. In the included BD studies, medication was usually reported but rarely controlled. In FVT studies, García-Blanco et al. (2014, 2017) [65,67] and Kjærstad et al. (2020) [70] found no significant medication effects in post hoc analyses, whereas most other studies reported medication only descriptively. Among saccadic studies, García-Blanco et al. (2013) [61] found that anxiolytic dose significantly predicted antisaccade latency, while Ritish et al. (2024) [60] found no association between eye-movement outcomes and either olanzapine-equivalent dose or lithium use; the findings of Dyer et al. (2026) [63] were broadly unchanged in medication-class sensitivity analyses. In SPEM studies, Holzman et al. (1991) found that lithium degraded pursuit integrity and increased saccadic intrusions, although its effect on gain was less consistent [78]. Brakemeier et al. (2020) found no associations with medication number or chlorpromazine-equivalent dose, and the findings persisted after excluding lithium-treated participants [79]. Limited analyses in the BR and EBR studies found no significant medication associations. However, these analyses were generally limited by small samples, polypharmacy, and incomplete dose reporting. Because psychotropic medications can affect saccadic latency and velocity, pursuit gain, blink rate, and alertness, residual medication confounding cannot be excluded. Future studies should report medication type, dose, duration, and timing and prespecify medication-adjusted or sensitivity analyses.

Eye-tracking equipment and sampling rates varied substantially across studies. FVT studies used systems ranging from 30 to 500 Hz, whereas saccadic studies used video-based systems operating at 250–1000 Hz or EOG recorded at 200 Hz. A 30 Hz system records one sample approximately every 33.3 ms, compared with 4 ms at 250 Hz, 2 ms at 500 Hz, and 1 ms at 1000 Hz. Lower sampling frequencies increase temporal uncertainty in measures such as event onset, latency, and duration, particularly when the observed effect approaches the interval between successive samples [94]. Brooks et al. similarly found that saccadic reaction times measured with a validated video system were 11 ms longer than those obtained with a reference eye tracker [95]. Such differences are important because antisaccade latency effects across psychiatric disorders are smaller and less robust than error-rate effects [96]. Importantly, the 30 Hz systems in the present review were used for FVT outcomes, including gaze duration, total fixation time, and fixation number, rather than for antisaccade latency or microsaccade measurements. Although the use of identical equipment supports within-study comparisons, differences in sampling rate, recording modality, calibration, filtering, and event-detection procedures limit cross-study comparability. Small differences in fixation, latency, velocity, or gain may therefore reflect methodological variation, precluding precise biomarker thresholds.

The risk-of-bias appraisal indicated that most studies clearly defined their samples and used objective diagnostic criteria; however, recurrent concerns included incomplete control of medication and other confounders, unclear reporting of measurement validity, and limitations in statistical analysis (Table S2). These weaknesses may have contributed to heterogeneity by producing study-specific associations or obscuring true group differences, thereby reducing confidence in the inconsistent findings across FVT, saccadic, and SPEM paradigms. Although BR findings were directionally more consistent, the small evidence base and unclear judgments in several appraisal domains limit confidence in their reproducibility. Accordingly, the available evidence supports preliminary group-level differences but not sufficiently robust evidence for clinical biomarker use.

This review has several limitations. First, the search was limited to PubMed and Scopus and to English-language studies, which may have excluded relevant studies indexed elsewhere. Second, because of substantial heterogeneity in paradigms, stimulus types, mood-state definitions, and outcome measures, quantitative synthesis was not performed. Third, most included studies were small and compared BD patients with HCs. Restricting the primary synthesis to separately extractable BD and BD-risk strata reduced diagnostic heterogeneity but may limit external validity because psychiatric comorbidities and diagnostic complexity are common in routine clinical practice. Consequently, the findings may not generalize fully to more representative BD populations, and the limited transdiagnostic evidence constrains conclusions about diagnostic specificity relative to MDD, schizophrenia-spectrum disorders, and other psychiatric conditions. Fourth, medication exposure, illness duration, psychotic features, BD subtype, and residual mood symptoms were inconsistently reported or controlled across studies. Finally, several paradigms, including MGS, vergence, and EBR, were represented by only one study, which limited the interpretation of their results. The review was not prospectively registered, and no protocol was published a priori, which limits transparency regarding whether methodological decisions were specified before study selection and synthesis.

## 5. Conclusions

Eye-tracking research in BD has focused primarily on attentional biases during FVTs and inhibitory control during saccadic tasks, with fewer studies examining SPEM, BR, MGS, vergence, and EBR. Findings were heterogeneous across FVT, saccadic, and SPEM studies, whereas BR showed consistency, reporting slower rivalry in at least one BD group. However, MGS, vergence, and EBR were each represented by only one study, and no measure demonstrated sufficient standardization, reproducibility, or individual-level validity for clinical biomarker use. The inconsistent results among the studies could be due to variations in study designs that included the severity of mood states, stimulus types and presentations, outcome measures, analytic methods, and targeted study subjects. Despite these limitations, eye-tracking paradigms can provide a powerful, non-invasive window into the neural systems underpinning affective, cognitive, and perceptual dysfunction in BD. The field needs standardized designs for different paradigms and prioritization of clinically relevant studies. To clarify the usefulness of eye-tracking technology in BD, future research should use standardized protocols, longitudinal designs, diagnostic comparison groups such as patients with MDD, and HR-BD or UHR-BD populations.

## Figures and Tables

**Table 1:** Methodological characteristics of free-viewing task studies in bipolar disorder.

Study	BD Type	Mood States	N	Stimulus Types	Paradigm	Source of Images	Primary Outcome Measures	Eye-Tracking Apparatus	Medication Status
Passive viewing of emotional images									
García-Blanco et al. (2014) [65]	BD-I	Mania (YMRS > 20) Depression (BDI-II > 18) Euthymia (BDI-II < 9, YMRS < 6) HC	23 20 23 20	Sad Happy Threatening Neutral	Four images simultaneously presented for 20 s	IAPS	Location of first fixation (%) Mean glance duration (ms) Percentage of fixation time (%) Percentage of fixations (%)	SMI RED250 Rate: 250 Hz	All patients were medicated: Li, antiepileptics, APs, ADs, anxiolytics
García-Blanco et al. (2015) [66]	BD-I	Mania (YMRS > 20) Depression (BDI-II > 18) Euthymia (BDI-II < 9, YMRS < 6) HC	26 24 26 23	Happy Threatening Neutral Control	¥ Two images (a happy/threatening/neutral and a control) simultaneously for 3 s	IAPS	Probability (%) and latency (ms) of first fixation Number of first-pass fixations (*n*) First-pass fixation duration (ms) Total fixation time (ms) Total fixation number (%)	SMI RED250 Rate: 250 Hz	All patients were medicated: Li, antiepileptics, APs, ADs, anxiolytics
Liu et al. (2019) [58]	BD-I	Mania (YMRS = 27.52 ± 3.28) HC	25 20	Sad Happy Neutral	Two facial images (a happy/sad and a neutral) for 5 s	CFAPS	Location/orientation of first fixation (%) Latency of first fixation (ms) Duration of first fixation (ms) Total fixation time (ms)	SMI RED500 Rate: 500 Hz	All patients were medicated: Li, VPA, olanzapine, quetiapine, chlorpromazine, risperidone
Peckham et al. (2016) [68]	BD-I	Euthymia, remitted (MHRSD < 7, YMRS < 7) HC	29 28	Sad Happy Fearful Neutral	Four facial expression images from same actor (sad/happy/fearful/neutral) for 10 s	MSFDE	Location of first fixation (%) Average duration (ms) Total fixation time (%)	Tobii T120 Rate: 120 Hz	77% of patients were medicated: Li, VPA, CBZ, lamotrigine, APs
AOI-based passive viewing of emotional scenes									
Purcell et al. (2018) [69]	BD-I	Euthymia, full or partial remission (IDS-C ≤ 11, YMRS ≤ 7) HC	24 25	Positive Negative Neutral	Single emotional images, for 5 s With AOI analysis	IAPS	Percentage of fixation time on emotional AOI (%) early (0.0–2.5 s), late (2.51–5.0 s), and total (0.0–5.0 s)	ASL Model 504 Rate: 60 Hz	Medication prevalence was not reported: Li, antiepileptics, neuroleptics, anxiolytics, stimulants, ADs, and sedative-hypnotics
Free viewing with instructional conditions									
Broch-Due et al. (2018) [71]	* BD	Euthymia, full or partial remission (HDRS-17 ≤ 14, YMRS ≤ 14)	15	Unpleasant Neutral	Single image (unpleasant/neutral) for 4 s 3 instructions: View Neutral, View Negative, and Dampen Negative	IAPS	Gaze duration (%) Total fixation time (%) Fixation number (*n*) AOI and whole-image analysis	Tobii X2-30 Rate: 30 Hz	Patients were medicated: Li, ADs, APs, antiepileptics, BDZs
		HC	16						
García-Blanco et al. (2017) [67]	BD-I	Mania (YMRS > 20) Depression (BDI-II > 18) Euthymia (BDI-II < 9, YMRS < 6) HC	29 27 28 27	Happy Threatening Neutral Control	¥ Two paired images (a happy, threatening, or neutral and a control) for 3 s Attend-to-emotional or Attend-to-neutral	IAPS	Latency of first fixation (ms) Percentage of first fixation (%) Gaze duration (ms) Total fixation time (ms)	SMI RED250 Rate: 250 Hz	Patients were medicated:Li, antiepileptics, APs, ADs, anxiolytics
Passive viewing of dynamic video clips									
Kjærstad et al. (2020) [70]	** BD-I/II	Euthymia, full or partial remission (HDRS-17 ≤ 14, YMRS ≤ 14) HC	38 40	Sad Happy Winning Racing Risk taking Socially Anxious Neutral	Passive viewing of 7 dynamic film clips lasting 90–110 s each, presented in random order The total duration was 13 min	Not applicable	Total gaze time (%) Total fixation time (%) Fixation number (*n*) AOI analysis	Tobii X2-30 Rate: 30 Hz	66% of patients were medicated:Li, APs, ADs, antiepileptics

Note: * BD subtype was not specified; ** BD-I (39.5%) and BD-II (60.5%) participants; ¥ Control images depicted inanimate/non-living objects, and neutral images depicted people engaged in non-emotional daily activities. Abbreviations: AD, antidepressant; AOI, area of interest; AP, antipsychotic; ASL, Applied Science Laboratories; BD, bipolar disorder; BD-I, bipolar disorder type I; BD-II, bipolar disorder type II; BDI-II, Beck Depression Inventory-II; BZD, benzodiazepine; CBZ, carbamazepine; CFAPS, Chinese Facial Affective Picture System; HC, healthy control; HDRS-17, Hamilton Depression Rating Scale; Hz, hertz; IAPS, International Affective Picture System; IDS-C, Inventory of Depressive Symptomatology-Clinician Rating; Li, lithium; MHRSD, Modified Hamilton Rating Scale for Depression; ms, milliseconds; MSFDE, Montreal Set of Facial Displays of Emotion; N, sample size; s, seconds; SMI, Senso Motoric Instruments; VPA, valproate; YMRS, Young Mania Rating Scale.

**Table 2:** Eye-tracking findings from free-viewing task studies in bipolar disorder.

Study	Initial Orienting		Attentional Engagement †	Sustained Attention (Overall Allocation of Attention)		Association with Symptom Severity
	Location of First Fixation *	Latency of First Fixation	Gaze Duration/First-Fixation or First-Pass Duration	Total Fixation Time/Percentage of Fixation Time	Total Fixation Number/Percentage of Fixations ‡	
García-Blanco et al. (2014) [65]	No difference between BD and HC. Happy > other images in all participants.	Not measured.	No difference between BD and HC. No difference for any emotional images.	D < HC for Happy. E/M/D > HC for Threat. No differences on Sad/Neutral.	D < HC for Happy. E/M/D > HC for Threat. No differences on Sad/Neutral.	Not measured.
García-Blanco et al. (2015) [66]	No difference between BD and HC. Happy/Threat > Neutral in all participants.	No difference between BD and HC. Happy/Threat < Neutral in all participants.	D > HC. Threat > Neutral in all participants.	No difference between BD and HC. Threat > Neutral in all BD states (E,M,D), but not for HC.	No difference between BD and HC. Threat > Neutral in all BD states (E,M,D), but not for HC. Happy > Neutral in Mania only.	BDI and first-pass fixation duration association on threat images (r = 0.22, *p* = 0.063).
Liu et al. (2019) [58]	M > HC on Neutral. No differences on Happy/Sad.	M > HC on overall images. No difference for any emotional images.	No difference between BD and HC. No difference for any emotional images.	M < HC overall. M < HC on Sad/Neutral. (No difference on Happy). Happy > Sad > Neutral in M. Happy/Sad > Neutral in HC.	Not measured.	Not measured.
Peckham et al. (2016) [68]	No difference between BD and HC. No difference for any emotional images.	Not measured.	No difference between BD and HC. Happy > Fear/Neutral > Sad in all participants.	No difference between BD and HC. Happy > Fear/Neutral > Sad in all participants.	Not measured.	MHRSD correlated with total (r = 0.39, *p* = 0.04) and average (r = 0.46, *p* = 0.02) fixation times to neutral.
Purcell et al. (2018) [69]	Not measured.	Not measured.	Not measured.	No difference between BD and HC. Neutral > Positive > Negative in all participants.	Not measured.	Not assessed as standalone correlations. Results remained null after controlling IDS-C and YMRS as covariates.
Broch-Due et al. (2018) [71]	Not measured.	Not measured.	Emotional reactivity: BD < HC on both Neutral/Negative images. Negative > Neutral for all participants. Emotional downregulation: BD < HC on all unpleasant pictures regardless of instruction (for whole-image, AOI showed only a trend). Dampening < passive viewing (for AOIs).	Emotional reactivity: No difference between BD and HC. No difference for any emotional images. Trend toward Negative > Neutral. Emotional downregulation: No difference between BD and HC (dampening vs. viewing negative) (for AOI/whole-image). Dampening < passive viewing (for AOIs).	Emotional reactivity: No difference between BD and HC. Negative > Neutral for all participants. Emotional downregulation: No difference between BD and HC (dampening vs. viewing negative) (for AOI/whole-image). Dampening < passive viewing (for AOIs).	↓ Gaze on neutral images correlated with depressive symptoms, r = –0.36, *p* = 0.049, and manic symptoms, r = –0.47, *p* = 0.007. ↓ Gaze on negative images correlated with manic symptoms, r = –0.43, *p* = 0.017.
García-Blanco et al. (2017) [67]	BD-HC difference depends on emotional images. M: Happy >Threat/Neutral HC: Threat > Neutral No difference for any emotional images in D and E. Attend-to-emotional > Attend-to-neutral in HC/E/M but not in D.	D/M > HC/E. Happy < Neutral/Threat in Attend-to-Neutral. No difference for any emotional images in Attend-to-Emotional block.	Attend-to-emotional: BD-HC difference depends on emotional images. M: Happy > Threat/Neutral D/E/HC: Neutral < Happy/Threat Attend-to-neutral: BD-HC difference depends on emotional images. M: Threat/Neutral > Happy D/E/HC: Neutral > Happy/Threat	Attend-to-emotional: HC≈E > M≈D Happy/Threatening > Neutral in all participants. Attend-to-neutral: BD-HC difference depends on emotional images. M: Threatening/Neutral > Happy D/E/HC: Neutral > Happy/Threat	Not measured.	YMRS scores were significantly correlated with the gaze duration for threatening scenes (r = 0.418, *p* <0.001).
Kjærstad et al. (2020) [70]	Not measured.	Not measured.	BD < HC overall (except risk-taking). (All group effects became NS after covariates.)	No difference between BD and HC.	No difference between BD and HC.	Subsyndromal depression: ↓ gaze time on Neutral, Sad, Racing, Happy, Socially Anxious. Subsyndromal mania: NS for all measures.

Note: ↓ indicates decreased gaze.* García-Blanco et al. (2015) [66] used probability of initial fixation, and García-Blanco et al. (2017) [67] used percentage of first fixation. Both refer to the proportion of first fixations directed to a target image or valence category. † García-Blanco et al. (2015) [66] reported first-pass fixation duration; Liu et al. (2019) [58] reported duration of first fixation; García-Blanco et al. (2017) [67] reported gaze duration in a first-pass sense; García-Blanco et al. (2014) [65] reported mean glance duration; Peckham et al. (2016) [68] reported average fixation duration; and Broch-Due et al. (2018) [71] and Kjærstad et al. (2020) [70] reported gaze-time measures closer to overall gaze allocation. ‡ Number of first-pass fixations was reported only by García-Blanco et al. (2015) [66]. This refers to the number of fixations during the first viewing episode and should not be equated with total fixation number or percentage of fixations. Abbreviations: AOI, area of interest; BD, bipolar disorder; BDI, Beck Depression Inventory; D, depressive state; E, euthymic state; HC, healthy control; IDS-C, Inventory of Depressive Symptomatology-Clinician Rating; M, manic state; MHRSD, Modified Hamilton Rating Scale for Depression; NS, not significant; YMRS, Young Mania Rating Scale.

**Table 3:** Methodological characteristics of saccadic paradigms in bipolar disorder.

Study	BD	Mood States	N	Stimulus Types	Source of Images	Paradigm	Primary Outcome Measures	Eye-Tracking Apparatus	Medication Status
Antisaccade with emotional images									
García-Blanco et al. (2013) [61]	BD-I	Mania (YMRS >20) Depression (BDI-II >18) Euthymia (BDI-II < 9 YMRS < 6) HC	22 25 24 28	Happy, Sad, Neutral	FACES database	AS and PS tasks (60 trials each, 20 per emotion) 1600 ms central fixation 1600 ms lateralized stimulus	Error rate (%) Latency of correct saccade (ms)	SMI RED250 Rate: 250 Hz	All BD patients were medicated: Li, antiepileptics, APs, ADs, anxiolytics.
Liu et al. (2019) [58]	BD-I	* Mania (YMRS = 27.5) HC	25 20	Happy, Sad, Neutral	CFAPS	Standard AS and PS tasks (60 trials each, 20 per emotion) 1000–1500 ms central fixation 1000 ms lateralized stimulus	Accuracy of the first correct saccade (%) Latency of the first correct saccade (ms)	SMI RED500 Rate: 500 Hz	All BD patients were medicated: Li, VPA, olanzapine, quetiapine, chlorpromazine, risperidone.
Antisaccade with non-emotional stimuli									
Dyer et al. (2026) [63]	BD-I	Euthymic (MADRS ≤ 12, YMRS ≤ 10) HC	44,108	Central fixation cross and peripheral circle targets	Not applicable	AS and PS task 6 blocks of 12 trials (36 gap and 36 step trials)	Error rate (%) Latency of correct saccade (ms)	EyeLink 1000 Rate: 500 Hz	79.5% of patients were medicated: Li, APs, ADs, MS, antiepileptics, BDZs.
Ritish et al. (2024) [60]	BD-I	FEM, remitted (HDRS < 8, YMRS < 8) HR-BD HC	31 3130	Targets on black background at ±6° and ±12° lateral positions (stimulus shape not specified)	Not applicable	AS and PS task 48 AS and 24 PS trials SPEM step-ramp task	Error rate (%) Mean first correct saccade peak velocity (°/s); latency (ms) Position error (°)	EyeLink 1000 Rate: 1000 Hz	All FEM patients were medicated: Li, APs.
Ekin et al. (2023) [59]		** UHR-BD HC HDRS YMRS	28 29	AS: Gray plus fixation mark and gray circle on black background MGS: Red plus fixation mark and red circle on black background	Not applicable	AS task (68 trials) 1000 ms fixation (gray plus) → 200 ms gap → target (gray dot) up to 1000 ms MGS task (48 trials) 1000 ms fixation (red plus) → 200 ms cue (red plus and dot) → 2000 ms delay (red plus) → up to 1000 ms response	Corrected saccades (*n*) Corrected errors (*n*) Uncorrected errors (*n*) Anticipatory saccades (*n*) Express saccades (*n*) Latency (ms), amplitude (°), peak velocity (°/s)	EyeLink 1000 Plus Rate: 1000 Hz	UHR-BD participants were medicated: MS, APs, ADs.
Antisaccade with a mixed saccadic task paradigm									
Beynel et al. (2014) [64]	BD	† Depression (MADRS > 20) HC	12 12	SPAN task (Saccade: Pro, Anti and No); AS, PS, no saccades (NS); 80 trials Cue: Fixation color (red: AS; green: PS; blue: NS) Peripheral cue and target (numbers)	Not applicable	Central fixation dot (white) → then became red, blue or green for 2 s to indicate AS, PS or NS → 200 ms gap → peripheral numeric cue for 50 ms → 1000 ms response window 500 ms central fixation	Error rates (%) Saccadic latency (ms)	EyeLink 1000 Rate: 500 Hz	BD participants received stable MS treatment for ≥4 weeks.
Dyer et al. (2025) [62]	BD	Euthymic (MADRS ≤ 12, YMRS ≤ 10) HC	44 30	Cold: Dots (neutral) Hot: Positive (joyful), negative (sad, fearful)	IAPS	Cold AS: neutral targets (circle), 72 trials in total, comprising 36 gaps and 36 step trials Hot AS: 72 trials in total, with 36 positive and 36 negative images for which each comprised 18 gap and 18 step trials Instruction cue (away/toward) Mixed block design with emotional valence (neutral, positive, negative) and attentional disengagement (step, gap)	Error rate (%) Latency of correct saccade (ms)	EyeLink 1000 Rate: 500 Hz	79.5% of patients were medicated: Li, APs, ADs, MS, antiepileptics, BDZs.
Prosaccade studies									
Velasques et al. (2013) [72]	BD	‡ Depression (YMRS = 4.66, CGI-BP-depression = 3.57) Mania (YMRS = 15.66, CGI-BP-depression = 1) HC	10 10 12	Light Emitting Diodes (LEDs)	Not applicable	Predictable PS task 12 blocks of 20 trials each	Saccade latency (ms)	EOG Rate: 200 Hz	All BD patients were medicated: ADs, MS.
Cartier et al. (2015) [20]	BD-I $	Depression Mania Euthymia	10 10 10	Light Emitting Diodes (LEDs)	Not applicable	Predictable PS task 12 blocks of 20 trials each	Saccade latency (ms)	EOG Rate: 200 Hz	Not reported.

Note: * Liu et al. (2019) [58] reported YMRS as mean ± SD, 27.52 ± 3.28, and did not provide an explicit manic-state cutoff; ** Ekin et al. (2023) [59] reported HDRS = 4.79 ± 4.18 and YMRS = 0.89 ± 2.38 for UHR-BD; HDRS = 0.34 ±1.14 and YMRS was not reported for HCs; † Beynel et al. (2014) [64]: BD subtype was reported as type I, II, or III; the definition of BD-III was not clearly specified in the primary paper; ‡ Velasques et al. (2013) [72] reported YMRS scores of 15.66 ± 7.00 for the manic group and 4.66 ± 3.96 for the depressed group. CGI-BP-depression version for both groups; $ Cartier et al. (2015) [20] diagnosed BD using SCID-I for DSM-IV and grouped patients according to CGI-BP; numerical CGI-BP values were not reported. Dyer et al. (2025) [62] and Dyer et al. (2026) [63] included the same 44 BD participants and overlapping cold antisaccade data; therefore, they were treated as linked reports rather than independent samples. The 2026 study included an expanded HC sample. Abbreviations: AD, antidepressant; AP, antipsychotic; AS, antisaccade; BD, bipolar disorder; BD-I, bipolar I disorder; BDI-II, Beck Depression Inventory-II; BDZ, benzodiazepine; CFAPS, Chinese Facial Affective Picture System; CGI-BP, Clinical Global Impression-Bipolar version; EOG, electrooculography; FEM, first episode mania; HC, healthy control; HDRS, Hamilton Rating Scale for Depression; HR-BD, high risk for bipolar disorder; Hz, hertz; IAPS, International Affective Picture System; LED, light emitting diode; Li, lithium; MADRS, Montgomery–Åsberg Depression Rating Scale; MGS, memory-guided saccade; ms, milliseconds; N, number; NS, no-saccade; PS, prosaccade; SMI, SensoMotoric Instruments; SPEM, smooth pursuit eye movement; UHR-BD, ultra-high risk for bipolar disorder; VPA, valproate; YMRS, Young Mania Rating Scale.

**Table 4:** Antisaccade error performance in bipolar disorder.

Outcome Measures	Study	Subjects	Percentage, Number, Emotional Stimulus				Patients vs. HCs
Percentage (%) of error rates	Dyer et al. (2025) [62] (Cold and Hot AS, step trials)		Neutral	Negative	Positive	* Emotionally charged overall	
		Euthymic	46.1 ± 27.0	35.2 ± 23.2	34.4 ± 23.0	34.4 ± 20.5	No significant difference
		HC	40.8 ± 22.7	34.3 ± 21.4	25.9 ± 19.3	30.2 ± 17.5	
			¥ Difference in % of errors between neutral and emotional stimuli				
			Neutral-Emotional	Neutral-Negative	Neutral-Positive	Negative-Positive	No significant difference
		Euthymic	11.7 ± 23.5	10.9 ± 24.6	11.8 ± 26.7	0.8 ± 20.1	
		HC	10.6 ± 21.4	6.5 ± 26.4	14.9 ± 20.9	8.5 ± 21.2	
	García-Blanco et al. (2013) [61]	Mania	47.7				M > HC, D > HC, M/D > E M: ↑ error in Happy (vs Sad or Neutral) D: trend toward ↑ error with Sad (vs. Neutral)
		Depression	32.5				
		Euthymic	13.3				
		HC	19.3				
	Ritish et al. (2024) [60]	FEM	≠81.6 (28.94–97.29)				*p* = 0.01 FEM > HC
		HR-BD	≠64.3 (19.14–97.14)				
		HC	≠50.6 (0–97.22)				
	Dyer et al. (2026) [63]	Euthymic	43.9 ± 25.29				No significant difference (*p* = 0.088)
		HC	36.56 ± 23.27				
Percentage (%) of accuracy of the first saccade			Neutral	Happy	Sad		
	Liu et al. (2019) [58]	Mania	62 ± 37	53 ± 32	57 ± 35		No group difference (*p* = 0.065) No expression difference (*p* = 0.690)
		HC	66 ± 34	77 ± 32	79 ± 21		
Corrected error counts (mean ± SD)	Ekin et al. (2023) [59]	UHR-BD	0.18 ± 0.48				No significant difference (*p* = 0.197 vs. *p* = 0.097)
		HC	0.83 ± 2.05				
Uncorrected error counts (mean ± SD)		UHR-BD	0.93 ± 1.15				
		HC	0.45 ± 0.78				

Note: ↑ indicates an increased error rate.* “Emotionally charged overall” refers to negative and positive stimuli combined, and all values are from the Supplementary Materials and represent step-trial error rates in the original publication; ¥ Scores represent the error rates for within-group differences with different valence contrasts; ≠ The values represent the median error rate and the range of error rate of each group. Abbreviations: AS, antisaccade; D, depressed bipolar disorder; E, euthymic bipolar disorder; FEM, first-episode mania; HC, healthy control; HR-BD, high risk for bipolar disorder; M, manic bipolar disorder; SD, standard deviation; UHR-BD, ultra-high risk for bipolar disorder.

**Table 5:** Antisaccade latency performance in bipolar disorder.

Outcome Measure		Study	Subjects	Mean ± SD or Median (Range) and Emotional Stimulus				Patients vs. HCs
Latency of Antisaccade eye movements (ms)	Mean ± SD	Dyer et al. (2025) [62] (Cold and Hot AS, step trials)		Neutral	Negative	Positive	* Emotionally charged overall	
			Euthymic	383.1 ± 65.2	369.1 ± 78.2	346.9 ± 50.4	360.8 ± 68.1	No significant difference
			HC	389.4 ± 63.2	374.6 ± 65.8	386.0 ± 96.7	377.6 ± 73.6	
				¥ Difference in latency between neutral and emotional stimuli				
				Neutral-Emotional	Neutral-Negative	Neutral-Positive	Negative-Positive	Negative-Positive was significant (*p* = 0.024), there was a trend for Neutral-Positive (*p* = 0.059), but others were not significant
			Euthymic	22.4 ± 77.6	14.7 ± 76.0	37.0 ± 66.4	22.2 ± 58.4	
			HC	11.8 ± 72.6	14.8 ± 69.9	3.4 ± 82.7	−11.5 ± 66.0	
		≠García-Blanco et al. (2013) [61]		Neutral	Happy	Sad		
			Mania	460 ± 66	478 ± 65	473 ± 58		D > HC M > HC E > HC
			Depression	470 ± 54	471 ± 73	465 ± 60		
			Euthymic	411 ± 59	412 ± 55	413 ± 57		
			HC	366 ± 47	370 ± 44	385 ± 52		
		£ Liu et al. (2019) [58]	Mania	452 ± 91	464 ± 86	468 ± 92		No group difference (*p* = 0.063) No expression difference (*p* = 0.406)
			HC	415 ± 74	426 ± 63	414 ± 66		
		Dyer et al. (2026) [63]	Euthymic	356.43 ± 56.22				No significant difference (*p* = 0.068)
			HC	339.25 ± 50.59				
		Ekin et al. (2023) [59]	UHR-BD	279.82 ± 55.1				No significant difference (*p* = 0.142)
			HC	256.11 ± 46.07				
	Median (Range)	$ Ritish et al. (2024) [60]	FEM	269.08 (135.0–438.55)				No significant difference (*p* = 0.65)
			HR-BD	259.4 (0.82–388·35)				
			HC	268.8 (175.25–358·00)				

Note: * “Emotionally charged overall” refers to negative and positive stimuli combined, and all values are from the Supplementary Materials and represent step-trial antisaccade latency in the original publication; ¥ Scores represent the antisaccade latency within-group differences with different valence contrasts; ≠ Mean corrected antisaccade latencies; £ Latency of the first correct saccade. $ The median time (ms) and the range of time (ms) of the antisaccade mean first correct saccade latency of each group. Abbreviations: AS, antisaccade; D, depressed bipolar disorder; E, euthymic bipolar disorder; FEM, first-episode mania; HC, healthy control; HR-BD, high risk for bipolar disorder; M, manic bipolar disorder; ms, milliseconds; SD, standard deviation; UHR-BD, ultra-high risk for bipolar disorder.

## Data Availability

No new data were created or analyzed in this study. Data sharing is not applicable to this article.

## Supplementary Materials

The following supporting information can be downloaded at: https://www.mdpi.com/article/10.3390/medicina62091626/s1, Table S1. Complete database-specific electronic search strategies; Table S2. Item-level risk-of-bias judgments for the included studies using design-appropriate revised JBI critical appraisal tools [20,37,58,59,60,61,62,63,64,65,66,67,68,69,70,71,72,75,76,77,78,79,81,82]; Table S3. Definition of attentional process during free-viewing task and contextual RDoC mapping [13].

## Abbreviations

The following abbreviations are used in this manuscript:

ADHD	Attention deficit hyperactivity disorder
AOI	Area of interest
AS	Antisaccade
ASL	Applied Science Laboratories
AUD	Alcohol use disorder
BD	Bipolar disorder
BD-I	Bipolar disorder type I
BD-II	Bipolar disorder type II
BDI	Beck Depression Inventory
BPwoP	Bipolar patients without psychotic features
BPwP	Bipolar patients with psychotic features
BR	Binocular rivalry
CFAPs	Chinese Facial Affective Picture System
DSM	Diagnostic and Statistical Manual of Mental Disorders
EBR	Eye-blink rate
EOG	Electrooculography
FEM	First episode mania
FVT	Free-viewing task
HC	Healthy control
HR	High risk
Hz	Hertz
IAPS	International Affective Picture System
ICD	International Classification of Diseases
iTBS	Intermittent theta burst stimulation
MADRS	Montgomery–Åsberg Depression Rating Scale
MDD	Major depressive disorder
MGS	Memory guided saccade
ms	Milliseconds
MSFDE	Montreal Set of Facial Displays of Emotion
NPC	Near point of convergence
OCD	Obsessive-compulsive disorder
PS	Prosaccade
RDoC	Research Domain Criteria
SANRA	Scale for the Assessment of Narrative Review Articles
SMI	SensoMotoric Instruments
SPAN	Saccade: Pro, Anti, No-saccade
SPEM	Smooth pursuit eye movement
UHR-BD	Ultra-high risk for bipolar disorder
YMRS	Young Mania Rating Scale
