# Supplementary material for: Eye-Tracking in Bipolar Disorder: A Methodology-Focused Narrative Review
Source: Medicina (Kaunas). 2026 Aug 24;62(9):1626. doi: 10.3390/medicina62091626 (PMC13608847; doi:10.3390/medicina62091626)
Supplement: Supplementary file 1 [file medicina-62-01626-s001.zip › medicina-4459272-supplementary.pdf]

Supplementary Materials

Table S1. Complete database-specific electronic search strategies

Panel A. PubMed		Panel B. Scopus	
Search characteristic	Details	Search characteristic	Details
Database	PubMed	Database	Scopus
Coverage	Database inception to 10 August 2026	Coverage	Database inception to 10 August 2026
Search fields	Medical Subject Headings (MeSH) and Title/Abstract	Search fields	Title, abstract, and keywords (TITLE-ABS-KEY)
Filters	No publication-date or language filters were applied during the database search	Filters	No publication-date or language filters were applied during the database search
Records retrieved	n = 367	Records retrieved	n = 745
Verbatim search strategy	("Bipolar Disorder"[Mesh] OR "bipolar disorder"[Title/Abstract] OR "bipolar depression"[Title/Abstract] OR "bipolar spectrum"[Title/Abstract] OR mania[Title/Abstract] OR manic[Title/Abstract] OR hypomani*[Title/Abstract]) AND ("Eye Movements"[Mesh] OR "eye tracking"[Title/Abstract] OR eyetrack*[Title/Abstract] OR "eye movement"[Title/Abstract] OR "eye movements"[Title/Abstract] OR oculomotor[Title/Abstract] OR "ocular motor"[Title/Abstract] OR saccad*[Title/Abstract] OR antisaccad*[Title/Abstract] OR prosaccad*[Title/Abstract] OR fixation*[Title/Abstract] OR gaze[Title/Abstract] OR scanpath*[Title/Abstract] OR "smooth pursuit"[Title/Abstract] OR vergence[Title/Abstract] OR "binocular rivalry"[Title/Abstract] OR "blink rate"[Title/Abstract] OR "eye blink"[Title/Abstract] OR "eye blinks"[Title/Abstract])	Verbatim search strategy	TITLE-ABS-KEY("bipolar disorder" OR "bipolar depression" OR "bipolar spectrum" OR mania OR manic OR hypomani*) AND TITLE-ABS-KEY("eye tracking" OR eyetrack* OR "eye movement" OR "eye movements" OR oculomotor OR "ocular motor" OR saccad* OR antisaccad* OR prosaccad* OR fixation* OR gaze OR scanpath* OR "smooth pursuit" OR vergence OR "binocular rivalry" OR "blink rate" OR "eye blink" OR "eye blinks")

Note: The searches retrieved 1,112 records before deduplication. After removal of 342 duplicate records, 770 unique records remained for screening. English-language eligibility was evaluated during screening rather than through a database filter.

Table S2. Item-level risk-of-bias judgments for the included studies using design-appropriate revised JBI critical appraisal tools.

Study	Q1	Q2	Q3	Q4	Q5	Q6	Q7	Q8
García-Blanco et al. (2014) [65]	Y	Y	Y	Y	Y	Y	Y	Y
García-Blanco et al. (2015) [66]	Y	Y	Y	Y	Y	N	Y	Y
Liu et al. (2019) [58]	Y	Y	U	Y	Y	N	Y	Y
Peckham et al. (2016) [68]	Y	Y	Y	Y	Y	N	Y	Y
Purcell et al. (2018) [69]	Y	Y	Y	Y	Y	N	Y	Y
Broch-Due et al. (2018) [71]	U	Y	Y	U	Y	N	U	Y
García-Blanco et al. (2017) [67]	Y	Y	Y	Y	Y	Y	Y	Y
Kjærstad et al. (2020) [70]	Y	Y	Y	U	Y	Y	N	Y
García-Blanco et al. (2013) [61]	Y	Y	Y	Y	Y	N	U	U
Ritish et al. (2024) [60]	Y	Y	U	Y	Y	N	U	Y
Ekin et al. (2023) [59]	Y	Y	Y	U	Y	N	N	Y
Beynel et al. (2014) [64]	U	U	U	Y	U	N	U	U
Dyer et al. (2025) [62]	U	Y	Y	U	Y	N	Y	U
Dyer et al. (2026) [63]	Y	Y	Y	U	Y	Y	N	Y
Velasques et al. (2013) [72]	Y	Y	U	U	Y	N	N	N
Cartier et al. (2015) [20]	Y	U	U	Y	N	N	N	N
Pettigrew and Miller (1998) [37]	Y	U	U	Y	Y	N	U	U
Nagamine et al. (2009) [75]	Y	Y	Y	Y	Y	Y	U	Y
Vierck et al. (2013)[76]	Y	U	U	Y	Y	Y	Y	Y
Law et al. (2017) [77]	Y	Y	Y	Y	Y	Y	Y	Y
Holzman et al. (1991) [78]	Y	Y	N	U	N	U	Y	Y
Brakemeier et al. (2020) [79]	Y	Y	Y	Y	Y	Y	U	Y
Chrobak et al. (2020) [81]	Y	Y	Y	Y	Y	Y	N	Y
Peckham and Johnson (2016) [82]	Y	Y	Y	Y	Y	Y	U	Y

Abbreviations: Y, yes; N, no; U, unclear; N/A, not applicable. No numerical summary scores or overall risk-of-bias classifications were calculated.

Table S3. Definition of attentional process during free-viewing task and contextual RDoC mapping

Metric	Attention Process	Unit	Definition
Location/probability/percentage of first fixation	Initial orienting	%	Proportion of trials in which the participant's first fixation landed on a target image or emotional valence category.
Latency of first fixation	Initial orienting	ms	Time from stimulus onset to the participant's first fixation on a target image.
Number of first-pass fixations	Attentional Engagement	n (%)	Number or percentage of fixations made on a target image during the first viewing episode, before gaze shifted away from that image.
First-pass fixation duration	Attentional Engagement	ms	Sum of fixation durations on a target image during the first viewing episode, before gaze shifted away from that image
Duration of first fixation	Attentional Engagement	ms	Duration of the first fixation made on a target image or emotional expression.
Mean glance duration	Attentional Engagement	ms	Average time gaze remained within the boundaries of an image or emotional valence category during a single glance, from gaze entry to gaze exit.
Average/mean duration of individual fixation	Attentional Engagement	ms	Average duration of individual fixations on a target image or emotional category across trials.
Gaze duration / gaze time	Attentional Engagement	ms/%	Total or average time gaze was directed toward a target image, AOI, or emotional category. Interpretation depends on the study definition; in some studies this reflects first-pass engagement, whereas in others it reflects overall gaze allocation.
Total fixation time / percentage fixation time	Sustained attention / overall allocation	ms or %	Sum or percentage of all fixation durations on a target image or AOI during the full stimulus exposure period, including re-fixations.
Total fixation number / percentage of fixations	Sustained attention / overall allocation	n/%	Number or percentage of all fixations and re-fixations directed to a target image or AOI during the full stimulus exposure period.

Note: Within the RDoC framework, the FVT metrics listed in this table are behavioral measures of the Attention construct within the Cognitive Systems domain. When emotional stimuli are used, Negative or Positive Valence Systems may also be engaged according to stimulus content; this contextual mapping applies to the paradigm rather than to individual eye-tracking metrics [13].
